# Tirzepatide versus other anti-diabetic agents for the prevention of diabetic foot complications in patients with type 2 diabetes

**DOI:** 10.3389/fphar.2026.1880132

**Published:** 2026-09-24

**Authors:** Chia-Yu Kuo, Jheng-Yan Wu, Ya-Wen Tsai, Chih-Cheng Lai, Jiun-Ling Wang

**Affiliations:** 1 Department of Anesthesiology, Chi Mei Medical Center, Tainan, Taiwan; 2 Department of Nutrition, Chi Mei Medical Center, Tainan, Taiwan; 3 Department of Public Health, College of Medicine, National Cheng Kung University, Tainan, Taiwan; 4 Division of Preventive Medicine, Chi Mei Medical Center, Tainan, Taiwan; 5 Department of Intensive Care Medicine, Chi Mei Medical Center, Tainan, Taiwan; 6 School of Medicine, College of Medicine, National Sun Yat-sen University, Kaohsiung, Taiwan; 7 Department of Internal Medicine, National Cheng Kung University Hospital, College of Medicine, National Cheng Kung University, Tainan, Taiwan

**Keywords:** amputation, complication, diabetes, diabetic foot, tirzepatide

## Abstract

**Background:**

Diabetic foot complications affect up to 25% of patients with type 2 diabetes (T2D) and represent a major cause of morbidity. The dual glucose-dependent insulinotropic polypeptide and glucagon-like peptide-1 receptor agonist - tirzepatide (TZP) has demonstrated superior glycemic control compared with semaglutide however, evidence regarding its effects on diabetic foot complications (DFCs) remains limited.

**Methods:**

We conducted a retrospective cohort study using the TriNetX network. Adults with T2D initiating TZP, sulfonylurea (SU), dipeptidyl peptidase-4 inhibitors (DPP-4is), sodium–glucose cotransporter-2 inhibitors (SGLT2is), and glucagon-like peptide-1 receptor agonists (GLP-1RAs) between January 2022 and July 2025 were included. Propensity score matching created balanced cohorts for four comparisons. The primary outcome was the risk of DFC - a composite of ulcers, infections, critical limb ischemia, and revascularization or amputation.

**Results:**

After matching, four cohorts included 141,002 patients per arm (TZP vs. SU), 131,449 patients per arm (TZP vs. DPP-4i), 146,321 patients per arm (TZP vs. SGLT2i), and 97,457 patients per arm (TZP vs. GLP-1RA). Compared with SU, TZP was associated with reduced diabetic foot complications (HR, 0.80; 95% CI, 0.76–0.84). Similar reductions were observed versus DPP-4i (HR, 0.83; 95% CI, 0.78–0.87) and SGLT2i (HR, 0.88; 95% CI, 0.83–0.92). No difference was found versus GLP-1RA (HR, 0.97; 95% CI, 0.91–1.03).

**Conclusion:**

TZP was associated with a reduction in diabetic foot complications compared with SU, DPP-4i, and SGLT2i, with outcomes similar to GLP-1RA. These findings suggest tirzepatide may be a valuable option for patients at risk of ulceration or amputation.

## Introduction

In 2021, an estimated 537 million people worldwide were living with diabetes mellitus (DM), a number projected to increase to 643 million by 2030 and 783 million by 2045 (Diabetes Federation, 2021). Among the many complications of diabetes, diabetic foot disorders, including ulceration, infection, ischemia, and gangrene, remain among the most debilitating, contributing substantially to morbidity, mortality, and healthcare costs (Armstrong et al., 2017; Zhang et al., 2020). Up to one-quarter of patients with DM will develop a foot ulcer in their lifetime, and such lesions are a leading cause of nontraumatic lower-extremity amputations (Singh et al., 2005). The pathogenesis of diabetic foot complications is multifactorial, involving peripheral neuropathy, peripheral arterial disease, impaired wound healing, and heightened susceptibility to infection (Armstrong et al., 2017; Boulton et al., 2005). Preventive strategies typically focus on intensive glycemic control, routine foot inspection, appropriate footwear, and early intervention for pre-ulcerative lesions (Singh et al., 2005; Bus et al., 2020). While these measures have reduced the incidence of severe events, residual risk remains high, particularly in those with long-standing or poorly controlled disease, highlighting the need for novel preventive therapies.

The dual glucose-dependent insulinotropic polypeptide and glucagon-like peptide-1 receptor agonist, tirzepatide (TZP), and glucagon-like peptide-1 receptor agonists (GLP-1RAs) belong to a class of incretin-based therapies that have demonstrated substantial benefits in glycemic control, weight reduction, and cardiometabolic outcomes in patients with type 2 diabetes (T2D) and obesity. In randomized trials, tirzepatide produced greater reductions in glycated hemoglobin and body weight than semaglutide, a well-established GLP-1RA (Fría et al., 2021; Aronne et al., 2025). Given this pharmacological distinction - GP/GLP-1 receptor agonism versus GLP-1 receptor agonism alone - the demonstrated differences in glycemic and weight outcomes between TZP and GLP-1RAs (Fría et al., 2021; Aronne et al., 2025), we analyzed TZP as a distinct exposure rather than pooling it with the broader GLP-1RA class, allowing us to isolate any incremental effect attributable to dual incretin-receptor engagement. Beyond glycemic control, tirzepatide exerts broad metabolic effects, including improvements in lipid profiles, blood pressure, and markers of inflammation, along with potential benefits for endothelial function and body fat distribution (Xia et al., 2024; Niu et al., 2024; Regmi et al., 2024; Forzano et al., 2022). Despite both GLP-1RAs and sodium–glucose cotransporter-2 inhibitors (SGLT2is) having demonstrated cardiovascular and kidney protection (Zelniker et al., 2019; Gerstein et al., 2019; Lin et al., 2022; Miceli et al., 2024), merging evidence suggests that incretin-based therapies, including tirzepatide, may also reduce the risk of major adverse limb events (MALEs) among individuals with diabetes mellitus (Go et al., 2025; Wu et al., 2025). However, whether tirzepatide provides similar protection against diabetic foot complications, including diabetic foot ulceration, infection, critical limb ischemia, or the need for lower extremity revascularization or amputation, remains uncertain.

To date, no large-scale real-world study has evaluated whether tirzepatide confers protection against diabetic foot ulceration, infection, critical limb ischemia, or the need for lower extremity revascularization or amputation. In this study, we compared the effectiveness of tirzepatide with that of three widely used classes of glucose-lowering agents, sulfonylurea (SU), dipeptidyl peptidase-4 inhibitors (DPP-4is), SGLT2is, and GLP-1RAs, in preventing diabetic foot complications among patients with T2D. We examined a comprehensive range of outcomes, including non–pressure and pressure ulcers, deep tissue infections such as abscess, cellulitis, and osteomyelitis, critical limb ischemia defined by peripheral arterial occlusive disease or gangrene, and subsequent limb procedures including revascularization and amputation. By evaluating a broad spectrum of clinically meaningful endpoints in a large, contemporary cohort with rigorous adjustment for baseline differences, this study aimed to determine whether tirzepatide offers incremental benefit for limb preservation, thereby informing therapeutic strategies to reduce the burden of diabetic foot disease.

## Methods

### Data source

This retrospective cohort study utilized TriNetX, a global federated health research network that aggregates de-identified electronic health records from approximately 182 million individuals across 155 healthcare organizations (HCOs) (Nassar et al., 2025). The database includes comprehensive clinical information such as diagnostic codes, procedures, prescribed medications, laboratory results, and genomic data. The study was approved by the Institutional Review Board of Chi Mei Hospital (IRB No. 11402-E02), which granted a waiver of informed consent given the retrospective design and use of anonymized data. Reporting adhered to the Strengthening the Reporting of Observational Studies in Epidemiology (STROBE) guidelines and the Reporting of studies Conducted using Observational Routinely-collected Health Data (RECORD) extension.

### Study design

Adults aged 18 years or older with a documented diagnosis of T2D (ICD-10-CM code E11) who initiated therapy with tirzepatide (TZP) or comparator antidiabetic agents, including SU, DPP-4i, SGLT2i or GLP-1RA between 1 January 2022, and 31 July 2025, were eligible. Only new users of these therapies were included, excluding individuals with prior or concurrent exposure to the study drugs before the index date. The index date was defined as the first recorded date of treatment initiation for each cohort. Patients were followed under an intention-to-treat approach, with follow-up time accrued from the index date regardless of subsequent treatment discontinuation, switching, or the addition of other antidiabetic agents. To assess the comparative effects of TZP, we conducted four separate head-to-head analyses: TZP versus SU, TZP versus DPP-4i, TZP versus SGLT2i, and TZP versus GLP-1RA. Definitions and coding algorithms for study variables are provided in Supplementary Table S1.

### Covariates and propensity score matching

Baseline characteristics were assessed during the 12 months before the index date. Propensity scores were calculated using logistic regression to estimate the likelihood of receiving each comparator therapy based on demographic, clinical, and treatment variables. Patients were matched one-to-one using nearest-neighbor matching with a caliper width of 0.1 of the standard deviation of the logit of the propensity score. Balance between matched cohorts was assessed using standardized mean differences (SMD), with values below 0.1 considered acceptable. Matching included age, sex, race/ethnicity, obesity defined as body mass index (BMI) ≥30 kg/m^2^, poor glycemic control defined as HbA1c ≥ 9%, low-density lipoprotein cholesterol, albumin, C-reactive protein, estimated glomerular filtration rate, comorbidities and the prevalence of T2D complications. Baseline medications comprised lipid-lowering drugs, antihypertensives, cardiovascular and antidiabetic agents, including background metformin use, which was captured and balanced as a covariate rather than analyzed as an independent exposure. Detailed coding for all covariates is provided in Supplementary Table S2.

### Outcomes

The primary outcome was diabetic foot complication (DFC), defined as a composite of non–pressure ulcers, pressure ulcers, abscesses, cellulitis, osteomyelitis, critical limb ischemia including peripheral artery occlusive disease, gangrene, and receipt of revascularization or amputation. Secondary outcomes included DFC with major adverse limb events (MALE), comprising peripheral artery occlusive disease, gangrene, and receipt of revascularization or amputation, as well as DFC without MALE, including non–pressure ulcers, pressure ulcers, abscesses, cellulitis, and osteomyelitis. To ensure ascertainment of incident events, patients with a diagnosis of any component outcome, including PAD, prior to or on the index date were excluded from the respective analysis. In addition, exploratory component-level analyses were conducted separately for non-pressure ulcer, pressure ulcer, cellulitis, abscess, osteomyelitis, peripheral arterial occlusive disease/critical limb ischemia, gangrene, revascularization, minor amputation, and major amputation. Follow-up began the day after the index date and continued until the first occurrence of any outcome, the last clinical encounter, death, or 1 year after the index date, whichever occurred first. Detailed definitions and coding algorithms for each outcome are provided in Supplementary Table S3.

### Statistical analysis

Continuous variables are reported as means with standard deviations, and categorical variables as counts and percentages. Propensity score matching (PSM) was performed before all primary and subgroup analyses to reduce confounding and improve comparability between treatment groups. Time-to-event outcomes were assessed using Cox proportional hazards models to estimate hazard ratios (HRs) with 95% confidence intervals (CIs). Kaplan–Meier curves and log-rank tests were used to compare event-free survival between groups. The proportional hazards assumption was evaluated using Schoenfeld residuals.

For predefined subgroup analyses, separate cohorts were re-created within TriNetX for each subgroup rather than analyzing subgroups within the original overall cohort. Predefined subgroup analyses were conducted by sex, age (18–64 vs. ≥65 years), race, and clinical characteristics, including the comorbidities and diabetic complications. Additional stratifications considered BMI (≥25 vs. <25 kg/m^2^) and eGFR (≥45 vs. <45 mL/min/1.73 m^2^). These subgroup analyses were performed to assess the consistency and robustness of the primary findings across clinically relevant patient subgroups and were not intended to formally evaluate treatment effect modification. Therefore, interaction P-values were not calculated. To assess the potential influence of unmeasured confounding, E-values were calculated to estimate the minimum strength an unmeasured confounder would need to fully explain the observed associations (VanderWeele and Ding, 2017). As a sensitivity analysis, in the TZP versus SU and TZP versus DPP-4i comparisons, patients who received an SGLT2i or GLP-1RA during the baseline period were excluded, after which PSM and outcome analyses were repeated. All analyses were conducted using the TriNetX analytics platform.

## Results

### Patients’ selection

The study utilized the TriNetX global federated health research database, containing 182,365,220 patients as of 03 September 2025. We extracted records for 7,062,806 patients diagnosed with T2D who had documented healthcare visits. Following the application of specified exclusion criteria, four comparison groups were established to evaluate TZP against SU, DPP-4i, SGLT2i, and GLP-1RA. To control for potential confounding variables, PSM was employed using a 1:1 ratio for each comparison group. The final matched cohorts consisted of: 141,002 patients per arm for the TZP versus SU comparison; 131,449 patients per arm for the TZP versus DPP-4i comparison; 146,321 patients per arm for the TZP versus SGLT2i comparison; and 97,457 patients per arm for the TZP versus GLP-1RA comparison (Figure 1).

**FIGURE 1:**
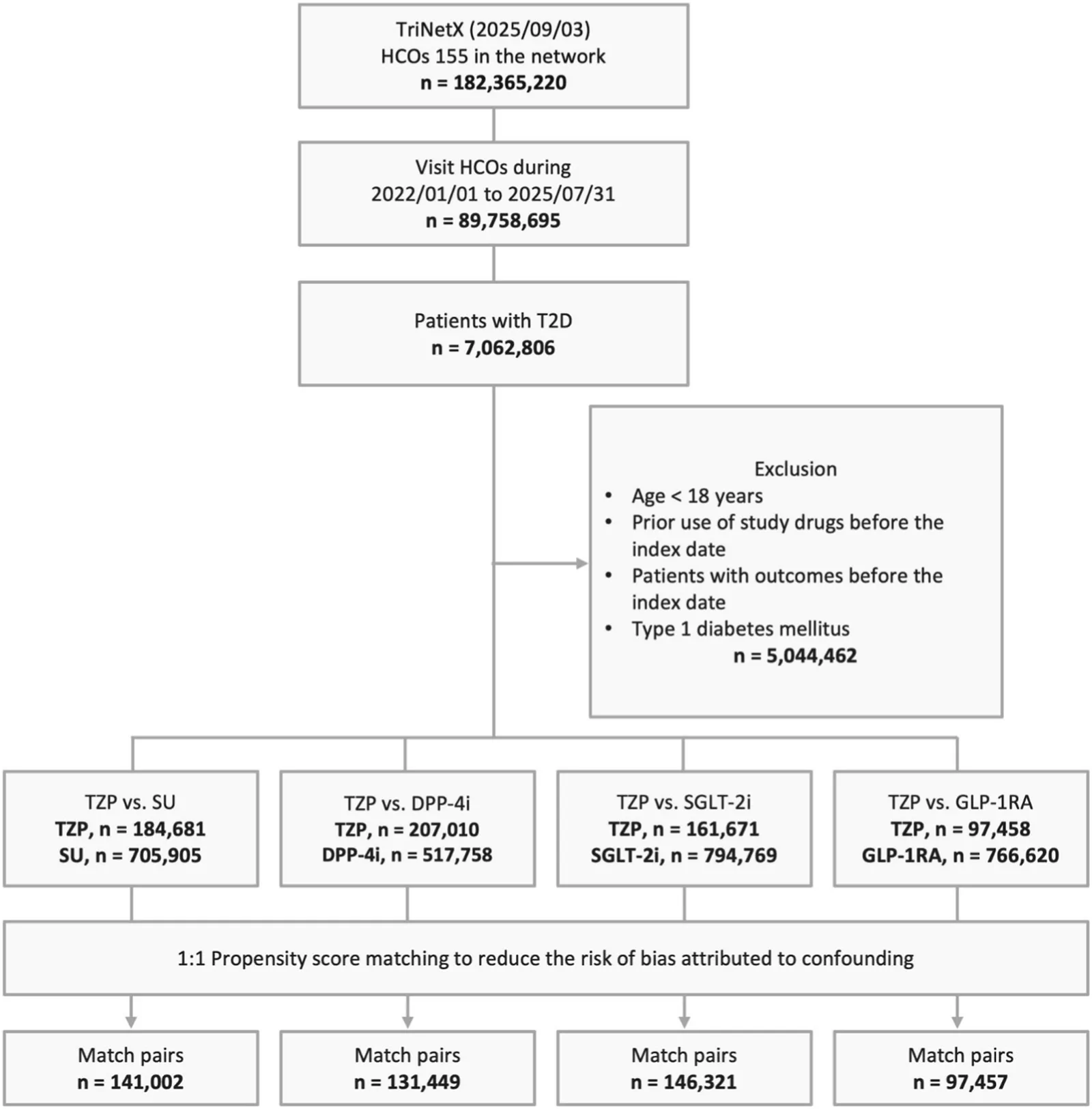
The algorithm for patient selection.

### Baseline characteristics

Before PSM, significant differences in baseline characteristics were found between TZP users and patients taking SU, DPP-4i, SGLT2i and GLP-1RA (Supplementary Table S4). After PSM, baseline characteristics were well-balanced across all comparison groups, including age, sex, race, BMI, HbA1c levels, comorbidities, T2D-related complications, and medications for cardiovascular disease, hypertension, and diabetes management. Balance was achieved in all four matched cohorts (TZP vs. SU; TZP vs. DPP-4i; TZP vs. SGLT2i; and TZP vs. GLP-1RA), with most SMD values < 0.1 (Table 1).

**TABLE 1:** Post-match baseline characteristics of tirzepatide and other antidiabetic medication cohorts.

Variables	TZP	SU	SMD	TZP	DPP-4i	SMD	TZP	SGLT-2i	SMD	TZP	GLP-1RA	SMD
Match pairs	n = 141,002			n = 131,449			n = 146,321			n = 97,457		
Age, years												
Mean (SD)	57.3 (12.3)	56.8 (13)	0.041	58.1 (12.2)	57.5 (12.9)	0.05	57.1 (12.4)	56.7 (12.7)	0.031	57.5 (12.6)	57.1 (12.9)	0.027
Sex, n (%)												
Female	76,721 (57)	77,915 (57.9)	0.018	73,785 (55.8)	74,871 (56.6)	0.017	89,626 (59.3)	90,357 (59.8)	0.01	53,872 (54.9)	54,879 (56)	0.021
Male	55,105 (40.9)	53,822 (40)	0.019	55,956 (42.3)	54,839 (41.5)	0.017	58,441 (38.7)	57,793 (38.2)	0.009	42,325 (43.2)	41,300 (42.1)	0.021
Race, n (%)												
White	89,978 (66.8)	91,965 (68.3)	0.032	87,332 (66.1)	89,210 (67.5)	0.03	100,757 (66.7)	101,880 (67.4)	0.016	67,457 (68.8)	67,218 (68.6)	0.005
Black or African American	22,208 (16.5)	22,363 (16.6)	0.003	22,289 (16.9)	22,948 (17.4)	0.013	26,322 (17.4)	26,652 (17.6)	0.006	15,751 (16.1)	16,066 (16.4)	0.009
Asian	4,982 (3.7)	4,302 (3.2)	0.028	5,170 (3.9)	4,348 (3.3)	0.033	4,880 (3.2)	4,572 (3)	0.012	3,327 (3.4)	3,314 (3.4)	0.001
Other race	5,354 (4)	5,129 (3.8)	0.009	4,995 (3.8)	4,876 (3.7)	0.005	5,920 (3.9)	5,730 (3.8)	0.007	3,673 (3.7)	3,553 (3.6)	0.006
Unknown race	10,318 (7.7)	9,061 (6.7)	0.036	10,742 (8.1)	9,068 (6.9)	0.048	11,328 (7.5)	10,466 (6.9)	0.022	6,686 (6.8)	6,732 (6.9)	0.002
Body mass index, kg/m^2^												
≥30, n (%)	91,792 (65.1)	92,779 (65.8)	0.014	78,738 (59.9)	80,315 (61.1)	0.024	99,498 (68.0)	99,791 (68.2)	0.004	65,881 (67.6)	65,394 (67.1)	0.012
Hemoglobin A1c, %												
≥9, n (%)	20,868 (14.8)	21,714 (15.4)	0.017	22,215 (16.9)	22,741 (17.3)	0.010	22,094 (15.1)	23,997 (16.4)	0.037	13,936 (14.3)	14,131 (14.5)	0.004
Comorbidities, n (%)												
Hypertension	78,622 (58.4)	76,741 (57)	0.028	77,147 (58.3)	75,661 (57.2)	0.023	89,118 (59)	88,801 (58.7)	0.004	56,581 (57.7)	55,511 (56.6)	0.022
Dyslipidemia	76,901 (57.1)	75,615 (56.2)	0.019	75,242 (56.9)	74,503 (56.3)	0.011	86,836 (57.4)	87,117 (57.6)	0.004	55,260 (56.4)	54,220 (55.3)	0.021
Chronic lower respiratory diseases	21,108 (15.7)	20,810 (15.5)	0.006	19,992 (15.1)	19,881 (15)	0.002	24,056 (15.9)	23,744 (15.7)	0.006	15,434 (15.7)	14,885 (15.2)	0.015
Ischemic heart disease	18,095 (13.4)	16,969 (12.6)	0.025	18,350 (13.9)	17,428 (13.2)	0.02	18,036 (11.9)	17,573 (11.6)	0.01	13,217 (13.5)	12,587 (12.8)	0.019
Neoplasms	22,952 (17)	22,129 (16.4)	0.016	22,057 (16.7)	21,618 (16.4)	0.009	26,622 (17.6)	25,769 (17)	0.015	16,744 (17.1)	16,158 (16.5)	0.016
Chronic kidney disease	13,774 (10.2)	13,072 (9.7)	0.017	14,559 (11)	13,841 (10.5)	0.018	14,372 (9.5)	14,420 (9.5)	0.001	10,113 (10.3)	9,679 (9.9)	0.015
Cerebrovascular diseases	5,735 (4.3)	5,234 (3.9)	0.019	6,013 (4.5)	5,685 (4.3)	0.012	5,955 (3.9)	5,862 (3.9)	0.003	4,205 (4.3)	3,967 (4)	0.012
Overweight and obesity	53,340 (39.6)	53,517 (39.7)	0.003	47,930 (36.3)	48,137 (36.4)	0.003	64,453 (42.6)	63,855 (42.2)	0.008	38,756 (39.5)	38,061 (38.8)	0.015
Obstructive sleep apnea	24,406 (18.1)	24,392 (18.1)	0	21,901 (16.6)	21,649 (16.4)	0.005	29,265 (19.4)	28,917 (19.1)	0.006	18,820 (19.2)	17,934 (18.3)	0.023
Heart failure	9,435 (7)	8,861 (6.6)	0.017	9,566 (7.2)	9,100 (6.9)	0.014	8,148 (5.4)	8,100 (5.4)	0.001	7,144 (7.3)	6,721 (6.9)	0.017
Atrial fibrillation and flutter	7,956 (5.9)	7,459 (5.5)	0.016	8,020 (6.1)	7,687 (5.8)	0.011	8,032 (5.3)	7,653 (5.1)	0.011	6,174 (6.3)	5,737 (5.9)	0.019
Nicotine dependence	8,996 (6.7)	8,733 (6.5)	0.008	8,664 (6.6)	8,840 (6.7)	0.005	9,812 (6.5)	9,645 (6.4)	0.005	6,577 (6.7)	6,392 (6.5)	0.008
Alcohol related disorders	1,870 (1.4)	1,691 (1.3)	0.012	1,785 (1.4)	1,773 (1.3)	0.001	2,002 (1.3)	1,952 (1.3)	0.003	1,479 (1.5)	1,430 (1.5)	0.004
Type 2 diabetes complications												
Kidney complications	13,205 (9.8)	12,745 (9.5)	0.012	13,843 (10.5)	13,265 (10)	0.014	13,946 (9.2)	14,148 (9.4)	0.005	9,170 (9.4)	8,876 (9.1)	0.01
Ophthalmic complications	5,293 (3.9)	5,045 (3.7)	0.01	5,602 (4.2)	5,366 (4.1)	0.009	5,811 (3.8)	6,015 (4)	0.007	3,314 (3.4)	3,138 (3.2)	0.01
Neurological complications	12,851 (9.5)	12,678 (9.4)	0.004	13,192 (10)	13,104 (9.9)	0.002	14,240 (9.4)	14,689 (9.7)	0.01	8,395 (8.6)	8,177 (8.3)	0.008
Circulatory complications	6,925 (5.1)	6,744 (5)	0.006	6,833 (5.2)	6,721 (5.1)	0.004	7,847 (5.2)	7,925 (5.2)	0.002	5,227 (5.3)	4,897 (5)	0.015
Medication, n (%)												
HMG CoA reductase inhibitors	57,532 (42.7)	56,676 (42.1)	0.013	57,351 (43.4)	56,814 (43)	0.008	66,098 (43.7)	66,425 (43.9)	0.004	41,572 (42.4)	40,430 (41.2)	0.024
Fibrates	4,111 (3.1)	4,027 (3)	0.004	4,236 (3.2)	4,161 (3.1)	0.003	4,258 (2.8)	4,335 (2.9)	0.003	2,871 (2.9)	2,614 (2.7)	0.016
Ezetimibe	4,176 (3.1)	4,041 (3)	0.006	4,172 (3.2)	4,102 (3.1)	0.003	4,625 (3.1)	4,484 (3)	0.005	3,185 (3.2)	3,042 (3.1)	0.008
Diuretics	36,077 (26.8)	35,604 (26.4)	0.008	35,705 (27)	35,018 (26.5)	0.012	41,497 (27.5)	40,969 (27.1)	0.008	26,494 (27)	25,440 (25.9)	0.024
Beta blockers related	33,145 (24.6)	32,120 (23.9)	0.018	33,259 (25.2)	32,437 (24.5)	0.014	36,299 (24)	35,525 (23.5)	0.012	24,359 (24.8)	23,238 (23.7)	0.027
Angiotensin II inhibitors	28,625 (21.3)	27,980 (20.8)	0.012	28,150 (21.3)	27,525 (20.8)	0.012	33,416 (22.1)	33,021 (21.8)	0.006	21,858 (22.3)	20,989 (21.4)	0.021
Angiotensin-converting enzyme inhibitors	25,000 (18.6)	24,885 (18.5)	0.002	25,402 (19.2)	25,492 (19.3)	0.002	28,492 (18.9)	29,071 (19.2)	0.01	17,626 (18)	17,285 (17.6)	0.009
Calcium channel blocker	24,623 (18.3)	23,740 (17.6)	0.017	25,336 (19.2)	24,456 (18.5)	0.017	29,054 (19.2)	28,733 (19)	0.005	18,824 (19.2)	17,962 (18.3)	0.023
Aspirin	14,788 (11)	14,137 (10.5)	0.016	15,387 (11.6)	14,484 (11)	0.022	15,090 (10)	14,874 (9.8)	0.005	10,374 (10.6)	9,928 (10.1)	0.015
Clopidogrel	4,330 (3.2)	4,115 (3.1)	0.009	4,659 (3.5)	4,472 (3.4)	0.008	4,086 (2.7)	4,030 (2.7)	0.002	3,240 (3.3)	3,161 (3.2)	0.005
Ticagrelor	504 (0.4)	464 (0.3)	0.005	567 (0.4)	520 (0.4)	0.006	451 (0.3)	477 (0.3)	0.003	362 (0.4)	315 (0.3)	0.008
Cangrelor	16 (0)	20 (0)	0.003	19 (0)	18 (0)	0.001	11 (0)	19 (0)	0.005	15 (0)	10 (0)	0.005
Prasugrel	278 (0.2)	273 (0.2)	0.001	304 (0.2)	291 (0.2)	0.002	263 (0.2)	269 (0.2)	0.001	226 (0.2)	230 (0.2)	0.001
Nitroglycerin	5,733 (4.3)	5,333 (4)	0.015	5,909 (4.5)	5,563 (4.2)	0.013	5,493 (3.6)	5,425 (3.6)	0.002	4,045 (4.1)	3,930 (4)	0.006
Ranolazine	486 (0.4)	503 (0.4)	0.002	491 (0.4)	497 (0.4)	0.001	408 (0.3)	404 (0.3)	0.001	358 (0.4)	377 (0.4)	0.003
Biguanides	51,877 (38.5)	52,484 (39)	0.009	51,411 (38.9)	51,942 (39.3)	0.008	58,774 (38.9)	60,211 (39.8)	0.019	37,728 (38.5)	37,497 (38.2)	0.005
Thiazolidinediones	3,144 (2.3)	3,121 (2.3)	0.001	3,780 (2.9)	3,856 (2.9)	0.003	3,576 (2.4)	3,648 (2.4)	0.003	2,492 (2.5)	2,451 (2.5)	0.003
Alpha glucosidase inhibitors	143 (0.1)	153 (0.1)	0.002	178 (0.1)	161 (0.1)	0.004	170 (0.1)	207 (0.1)	0.007	109 (0.1)	115 (0.1)	0.002
Insulins and analogues	30,762 (22.8)	30,236 (22.5)	0.009	31,349 (23.7)	31,204 (23.6)	0.003	32,992 (21.8)	34,714 (23)	0.027	19,051 (19.4)	18,447 (18.8)	0.016
Sulfonylureas	-	-	-	15,375 (11.6)	15,015 (11.4)	0.009	14,730 (9.7)	15,271 (10.1)	0.012	41,572 (42.4)	40,430 (41.2)	0.024
Dipeptidyl peptidase 4 inhibitors	14,233 (11.6)	13,955 (11.4)	0.007	-	-	-	18,672 (15.2)	18,513 (15.1)	0.004	5,945 (6.1)	5,779 (5.9)	0.007
Sodium-glucose co-transporter 2 inhibitors	20,820 (15.5)	20,806 (15.5)	0	20,194 (15.3)	19,838 (15)	0.008	-	-	-	10,018 (10.2)	9,811 (10)	0.007
Glucagon-like peptide-1 analogues	37,563 (27.9)	35,343 (26.2)	0.037	26,553 (20.1)	23,381 (17.7)	0.061	55,230 (36.5)	55,269 (36.6)	0.001	-	-	-
Laboratory data												
LDL-C, mg/dL	90.9 ± 37.8	91.9 ± 38.6	0.025	89.8 ± 38.2	90.4 ± 38.1	0.014	91.3 ± 37.7	89.1 ± 37.9	0.057	92.5 ± 38.1	91.7 ± 37.7	0.022
Albumin, g/dL	4.1 ± 0.4	4.1 ± 0.5	0.170	4.1 ± 0.4	4.0 ± 0.5	0.185	4.1 ± 0.4	4.1 ± 0.5	0.041	4.1 ± 0.4	4.1 ± 0.5	0.016
C-reactive protein, mg/L	24.4 ± 48.5	36.7 ± 60.9	0.224	25.9 ± 50.7	39.8 ± 64.8	0.239	24.3 ± 48.3	29.5 ± 54.5	0.101	25.9 ± 49.7	26.1 ± 48.7	0.004
eGFR, mL/min/1.73 m^2^	80.5 ± 26.8	81.3 ± 30.9	0.021	79.5 ± 27.2	78.6 ± 31	0.029	81 ± 26.7	81.2 ± 28.7	0.007	80.4 ± 27.1	80 ± 27.4	0.015

Baseline characteristics and medication use were assessed during the 12 months before the index date. Medication use reflects baseline exposure rather than confirmed concomitant therapy. DPP-4i, dipeptidyl peptidase 4 inhibitor; eGFR, estimated Glomerular filtration rate; GLP-1RA, glucagon-like peptide-1, receptor agonist; LDL-C, low-density lipoprotein cholesterol; SGLT2i, sodium-glucose cotransporter 2 inhibitor; SMD, standardized mean difference SU, sulfonylurea; TZP, tirzepatide.

### Primary outcome

#### TZP vs. SU cohort

Compared with SU, the TZP group was associated with the lower risk of DFC (1.6 per 100 person-years vs. 2.8 per 100 person-years; HR, 0.80; 95% CI, 0.76–0.84; p < 0.0001; Schoenfeld residual tests P value >0.05, Table 2). The E-value was 1.81 (95% LCL, 1.67). The Kaplan–Meier time-to-event curves showed the lower risk of DFC between patients receiving TZP and those receiving SU (log-rank p < 0.0001; Figure 2A). Further stratified analysis demonstrated consistent trends, with significant differences across most subgroups (Figure 3A).

**TABLE 2:** Hazard ratio of primary and secondary outcomes between tirzepatide and other comparator groups.

Comparator	Events (n)	Incidence rate per 100 PYs	Events (n)	Incidence rate per 100 PYs	HR (95% CI)	*P* value	E-value (95% LCL)
Primary outcome: diabetic foot complication							
Tirzepatide (n = 141,002) vs. SU (n = 141,002)	2,235	1.6	3,912	2.8	0.80 (0.76,0.84)	<0.0001	1.81 (1.67)
Tirzepatide (n = 131,449) vs. DPP-4i (n = 131,449)	2,152	1.6	3,726	2.8	0.83 (0.78,0.87)	<0.0001	1.70 (1.56)
Tirzepatide (n = 146,321) vs. SGLT-2i (n = 146,321)	2,412	1.6	3,513	2.4	0.88 (0.83,0.92)	<0.0001	1.53 (1.39)
Tirzepatide (n = 97,457) vs. GLP1-RA (n = 97,457)	1,665	1.7	2,284	2.3	0.97 (0.91,1.03)	0.1654	1.21 (1.00)
Secondary outcomes: diabetic foot complication without major adverse limb events							
Tirzepatide (n = 141,002) vs. SU (n = 141,002)	2,100	1.5	3,729	2.6	0.79 (0.75,0.83)	<0.0001	1.85 (1.70)
Tirzepatide (n = 131,449) vs. DPP-4i (n = 131,449)	2,075	1.6	3,402	2.6	0.85 (0.81,0.90)	<0.0001	1.63 (1.46)
Tirzepatide (n = 146,321) vs. SGLT-2i (n = 146,321)	2,246	1.7	3,218	2.4	0.90 (0.85,0.95)	0.0001	1.46 (1.29)
Tirzepatide (n = 97,457) vs. GLP1-RA (n = 97,457)	1,540	1.6	2,078	2.1	0.98 (0.92,1.05)	0.5078	1.16 (1.00)
Secondary outcomes: diabetic foot complication with major adverse limb events							
Tirzepatide (n = 141,002) vs. SU (n = 141,002)	1,702	1.2	2,688	1.9	0.79 (0.74,0.84)	<0.0001	1.85 (1.67)
Tirzepatide (n = 131,449) vs. DPP-4i (n = 131,449)	1,611	1.2	2,595	2.0	0.77 (0.73,0.82)	<0.0001	1.92 (1.74)
Tirzepatide (n = 146,321) vs. SGLT-2i (n = 146,321)	1,805	1.2	2,621	1.8	0.81 (0.76,0.86)	<0.0001	1.77 (1.60)
Tirzepatide (n = 97,457) vs. GLP1-RA (n = 97,457)	1,409	1.4	1,784	1.8	0.96 (0.90,1.03)	0.2774	1.25 (1.0)

CI, confidence interval; DPP-4i, dipeptidyl peptidase 4 inhibitor; GLP-1RA, glucagon-like peptide-1, receptor agonist; HR, hazard ratio; LCL, lower confidence limit; SU, sulfonylurea; SGLT-2i, sodium-glucose cotransporter 2 inhibitor.

**FIGURE 2:**
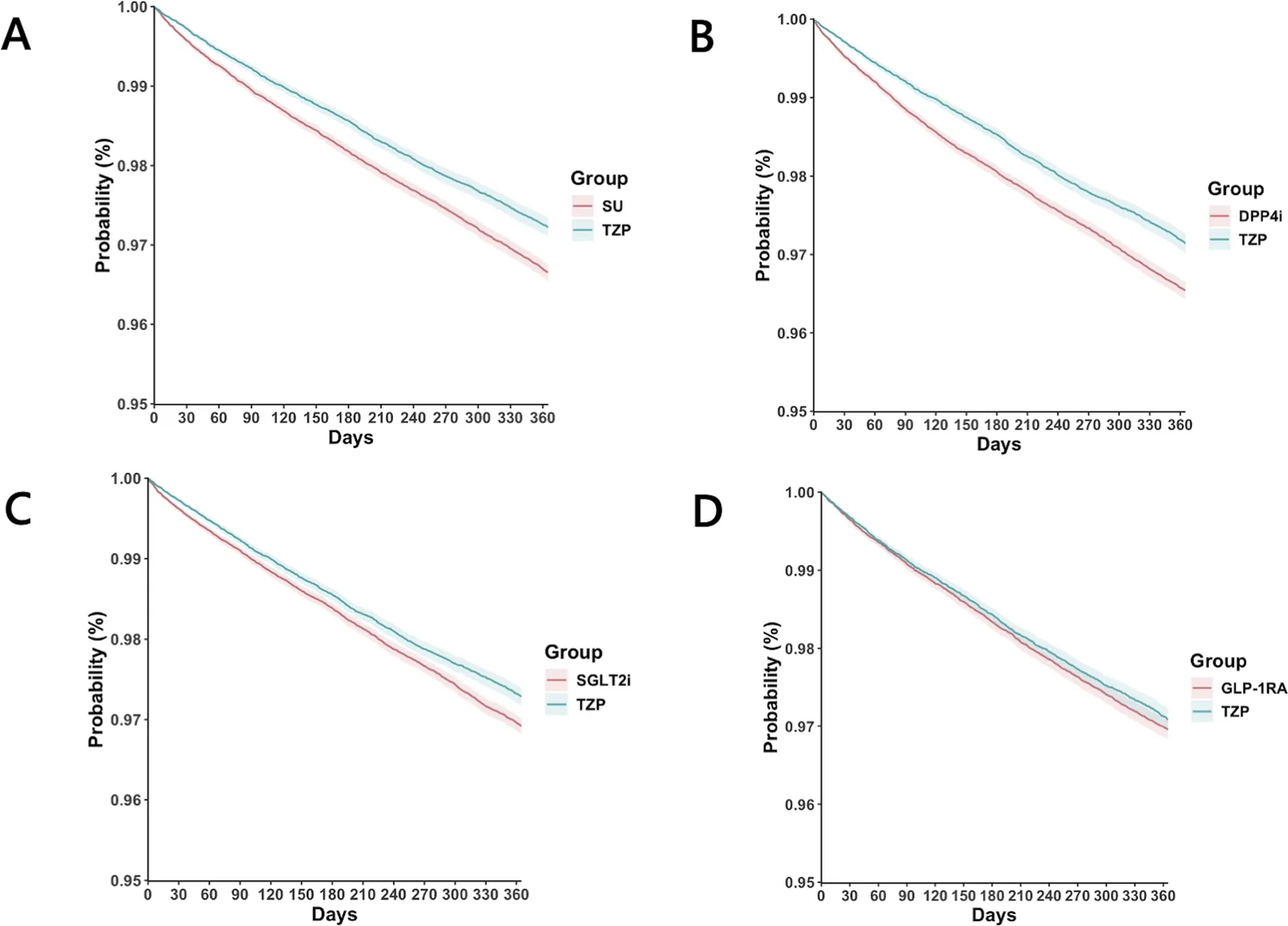
Kaplan-Meier curves showing time to even free of the primary outcome for tirzepatide (TZP) compared with sulfonylureas (SU) **(A)**, DPP-4 inhibitors (DPP-4i) **(B)**, SGLT2 inhibitors (SGLT2i) **(C)**, and GLP-1 receptor agonists (GLP-1RA) **(D)**.

**FIGURE 3:**
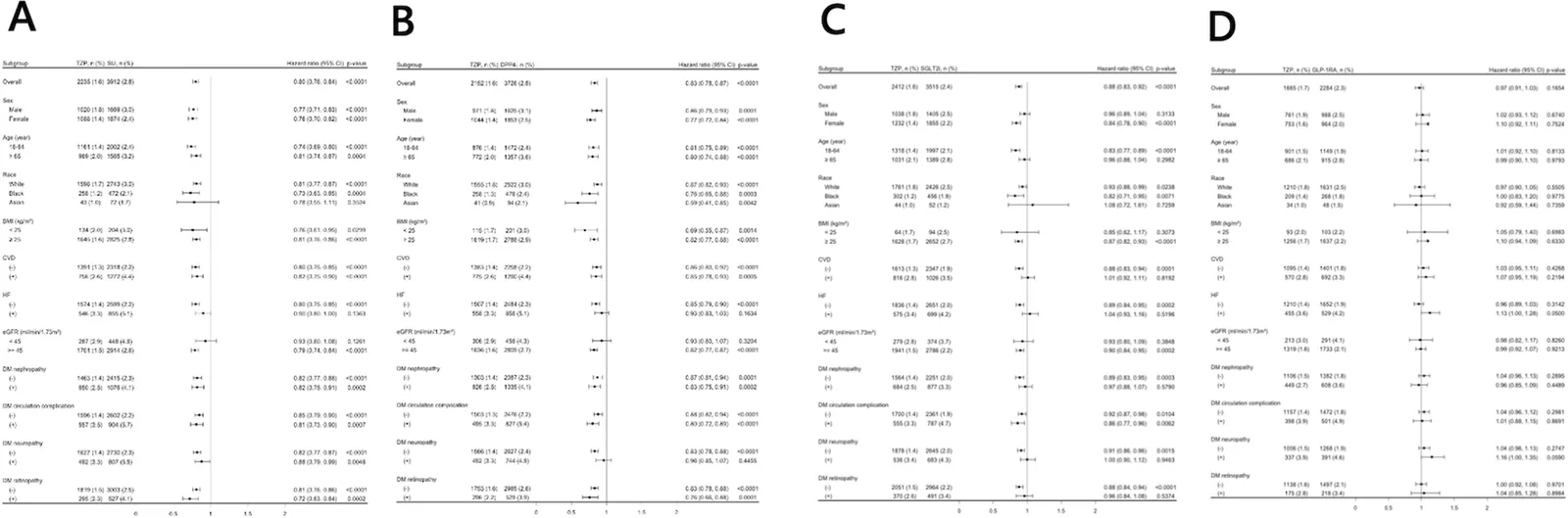
Stratified analysis of the risk of the primary outcome comparing tirzepatide (TZP) with **(A)** sulfonylureas (SU), **(B)** DPP-4 inhibitors (DPP-4i), **(C)** SGLT2 inhibitors (SGLT2i), and **(D)** GLP-1 receptor agonists (GLP-1RA). Abbreviations: BMI, body mass index; CI, confidence interval; CVD, cardiovascular disease; DM, diabetes mellitus; eGFR, estimated glomerular filtration rate; GLP-1RA, glucagon-like peptide-1 receptor agonist; HF, heart failure; HR, hazard ratio.

#### TZP vs. DPP-4i cohort

In the comparison with DPP-4i, TZP was associated with a decreased risk of DFC (1.6 per 100 person-years vs. 2.8 per 100 person-years; HR, 0.83; 95% CI, 0.78–0.87; p < 0.0001; Schoenfeld residual tests P value >0.05, Table 2). The E-value was 1.70 (95% LCL, 1.56). Kaplan–Meier survival estimates revealed a lower DFC risk for TZP compared with DPP-4i (log-rank p < 0.0001; Figure 2B). Results from stratified analyses demonstrated consistent findings, with significant differences in most subgroups (Figure 3B).

#### TZP vs. SGLT2i cohort

Relative to SGLT2i, TZP treatment was also linked to a reduced risk of DFC (1.6 per 100 person-years vs. 2.4 per 100 person-years; HR, 0.88; 95% CI, 0.83–0.92; p < 0.0001; Schoenfeld residual tests P value >0.05, Table 2). The E-value was 1.53 (95% LCL, 1.39). Time-to-event analyses confirmed the lower DFC risk with TZP compared with SGLT2i (log-rank p < 0.0001; Figure 2C). The stratified results further supported these associations, showing consistency across the majority of subgroups (Figure 3C).

#### TZP vs. GLP-1RA cohort

By contrast, no significant difference in DFC risk was observed between TZP and GLP-1RA user (1.7 per 100 person-years vs. 2.3 per 100 person-years; HR, 0.97; 95% CI, 0.91–1.03; p = 0.165; Schoenfeld residual tests P value >0.05, Table 2). The E-value was 1.21 (95% LCL, 1.00). Kaplan–Meier curves indicated comparable outcomes between the two groups (log-rank p = 0.165; Figure 2D). Subgroup analyses yielded broadly consistent patterns, without significant differences across categories (Figure 3D).

### Absolute risk reduction and number needed to treat

One-year absolute risk reduction analyses demonstrated clinically meaningful absolute benefits of tirzepatide (Supplementary Table S5). Compared with sulfonylureas, DPP-4 inhibitors, and SGLT2 inhibitors, tirzepatide achieved absolute risk reductions of 1.12%, 1.19%, and 0.75%, corresponding to NNTs of 89 (95% CI, 81–98), 84 (95% CI, 77–93), and 133 (95% CI, 118–154), respectively. In addition, the absolute risk reduction compared with GLP-1 receptor agonists was 0.63%, corresponding to an NNT of 158 (95% CI, 132–197). Sensitivity analyses excluding SGLT2i or GLP-1RA use yielded results consistent with the primary analyses, with lower risks of DFC observed for TZP versus SU (HR, 0.76; 95% CI, 0.72–0.81) and DPP-4i (HR, 0.83; 95% CI, 0.78–0.88) (Supplementary Table S7).

### Secondary outcomes

Regarding secondary outcomes (Table 2), TZP was associated with a lower risk of DFC without MALE compared with SU (HR, 0.79; 95% CI, 0.75–0.83), DPP-4i (HR, 0.85; 95% CI, 0.81–0.90), and SGLT2i (HR, 0.90; 95% CI, 0.85–0.95). TZP also showed a reduced risk of DFC with MALE compared with SU (HR, 0.79; 95% CI, 0.74–0.84), DPP-4i (HR, 0.77; 95% CI, 0.73–0.82), and SGLT2i (HR, 0.81; 95% CI, 0.76–0.86). In contrast, no significant differences were observed between TZP and GLP-1RA for either DFC without MALE (HR, 0.98; 95% CI, 0.92–1.05) or DFC with MALE (HR, 0.96; 95% CI, 0.90–1.03). Component-level analyses generally showed findings consistent with the composite outcomes (Supplementary Table S6). Compared with SU, DPP-4i, and SGLT2i, TZP was associated with lower risks across most individual components, including ulcer-related, infectious, vascular, and amputation outcomes. In contrast, most component-specific associations between TZP and GLP-1RA were not statistically significant, although TZP was associated with a lower risk of revascularization (HR, 0.69; 95% CI, 0.54–0.88). The proportional hazards assumption was satisfied for all Cox models, with all Schoenfeld residual tests yielding P values > 0.05.

## Discussion

In this large real-world cohort of patients with T2D, treatment with TZP was associated with significantly lower rates of DFC and related limb complications than SU, DPP-4i, or SGLT2i, whereas outcomes were equivalent to those seen with GLP-1RAs. Specifically, the HRs for the primary composite of foot ulcer/infection with TZP versus SU, DPP-4i, and SGLT2i were approximately 0.80, 0.83, and 0.88, respectively, reflecting a 12%–20% relative risk reduction. In contrast, TZP versus GLP-1RA yielded an HR of about 0.97 (95% CI 0.91–1.03), indicating no significant difference between these incretin-based therapies. Secondary analyses showed a similar pattern: TZP significantly reduced the risk of foot ulceration (with or without subsequent limb events) compared with DPP-4i, and SGLT2i, but not compared with GLP-1RAs. These findings add to emerging evidence that incretin therapies afford vascular protection in diabetes. These findings add to evidence that incretin-based therapies may provide vascular protection in diabetes and suggest that tirzepatide could be considered in patients at high risk for diabetic foot complications.

The comparative pattern observed across comparators is biologically plausible. TZP and GLP-1RAs share a common GLP-1 receptor–mediated mechanism, which likely accounts for their similar effects on the vascular, anti-inflammatory, and weight-related pathways implicated in DFC; the additional GIP receptor agonism of TZP appears to confer incremental metabolic bnefit (Fría et al., 2021; Aronne et al., 2025), but this may not translate into a further reduction in a downstream, multifactorial outcome such as DFC once GLP-1 receptor activity is already present. In contrast, SU and DPP-4i lack meaningful weight-lowering or vascular-protective effects, and SU carries an inherent risk of hypoglycemia, which may independently impair wound healing and increase susceptibility to falls and tissue injury. SGLT2i confer cardiorenal and modest weight benefits but act through mechanisms distinct from incretin signaling, particularly with respect to inflammation and endothelial function, which may explain the intermediate but still significant benefit observed for TZP relative to this comparator. Taken together, these comparative findings suggest that the magnitude of benefit associated with TZP may track more closely with a comparator’s incretin-pathway engagement than with glycemic efficacy alone.

Previous studies have demonstrated that GLP-1RA therapy was associated with greater major amputation-free survival compared with SGLT2is in patients with T2D. In our study, we further found that TZP provided additional preventive benefits against DFC when compared with DPP-4i, SGLT2i, and SU. Emerging evidence indicates that TZP may provide significant protection against MALEs among individuals with T2D (Go et al., 2025; Wu et al., 2025). This finding extends beyond glycemic control and weight reduction, highlighting a potential role of TZP in mitigating severe diabetes-related vascular and limb complications. Our study builds upon prior findings by showing that TZP not only reduces the risk of MALE but also provides protection against DFC. This suggests that TZP may exert broader benefits on diabetes-related lower extremity outcomes beyond limb salvage.

The pathophysiology of DFC involves several mechanisms, including peripheral neuropathy, vascular insufficiency with resultant ischemia, and secondary infection with associated inflammation. These overlapping pathways indicate that DFC result not only from mechanical injury but also from impaired tissue perfusion and host defense. In this context, TZP may provide protection through multiple mechanisms: potential vascular benefits that mitigate ischemia (Baser et al., 2024; Stefanou et al., 2024), anti-inflammatory effects (Xia et al., 2024; Borlaug et al., 2025), and metabolic actions that may reduce susceptibility to infection. TZP has been shown to significantly reduce ICAM-1 levels compared with placebo and to lower hs-CRP relative to baseline and placebo (Wilson et al., 2022). GLP-1RAs have demonstrated protective effects against inflammatory skin diseases, including psoriasis and hidradenitis suppurativa (Paschou et al., 2025), and because TZP combines GLP-1 and GIP receptor activity, it may also confer protective effects against skin inflammation. Clinical trials addressing this hypothesis are ongoing (Atiquzzaman et al., 2025; Morrissette et al., 2024). In addition, TZP has been shown to ameliorate high glucose–induced neurodegeneration and overcome neuronal insulin resistance, suggesting a potential role in improving diabetes-related neuropathy (Fontanella et al., 2024). Overall, these findings indicate that TZP exerts protective effects on vascular function, neuronal integrity, and inflammatory pathways. These pleiotropic actions of TZP are particularly relevant to the pathogenesis of DFC, which involve ischemia, neuropathy, and infection-driven inflammation. By addressing these interconnected mechanisms, TZP may provide protection beyond weight loss and glycemic control. Our findings extend prior evidence and support a potential role for TZP in mitigating the development and progression of DFC.

Obesity is an independent risk factor for infectious complications (Hu et al., 2022), with effects extending beyond those of hyperglycemia. Even after adjustment for glycemic control, obesity has been associated with higher rates of skin and soft tissue infections, osteomyelitis, and necrotizing fasciitis (Yang et al., 2021), and higher BMI has been linked to an increased risk of DFC (Sohn et al., 2011). The mechanisms underlying these associations may include impaired immune function, altered skin barrier integrity, and reduced tissue perfusion. TZP has demonstrated robust effects on weight reduction, which may in turn lower the risk of skin and soft tissue infections, osteomyelitis, and DFC. Furthermore, TZP treatment has been shown to reduce infiltration of pro-inflammatory M1 macrophages in adipose tissue and to decrease levels of inflammatory cytokines (Xia et al., 2024). Taken together, these observations suggest that the weight-lowering and anti-inflammatory effects of TZP may further contribute to reducing the burden of DFC. Thus, by simultaneously targeting metabolic, vascular, neuronal, and inflammatory pathways, TZP may provide a comprehensive therapeutic approach to the prevention and progression of DFC.

This study has notable strengths. We used a large, multinational electronic health record network (TriNetX) (Nassar et al., 2025), to construct well-balanced propensity-matched cohorts of approximately 100,000 patients for each comparison. Matching achieved excellent covariate balance, with most SMDs below 0.1. A broad spectrum of clinically meaningful outcomes was assessed, including non–pressure ulcers, deep tissue infections, critical limb ischemia, revascularization, and amputation, and the findings were consistent across sensitivity analyses. The inclusion of diverse health systems enhances the generalizability of the results and reflects outcomes observed in routine clinical practice.

Several limitations warrant consideration. First, despite rigorous PSM, residual confounding and selection bias cannot be excluded, particularly confounding by indication. Patients selected for TZP by their treating physicians may systematically differ from those started on SU, DPP-4i, or SGLT2i in ways not fully captured by our covariates, including a greater burden of obesity, more frequent specialist or endocrinology involvement, more contemporary diabetes management practices, higher socioeconomic status, and more frequent follow-up with access to multidisciplinary diabetic foot care. These factors independently influence foot outcomes and could not be fully balanced through matching. We calculated E-values to gauge the robustness of our findings to unmeasured confounding, and these were relatively high across primary comparisons; nevertheless, given the plausibility of substantial confounding by indication in this setting, the associations reported here should be interpreted as hypothesis-generating rather than causal, and the possibility that they partly reflect underlying differences in patient selection and care intensity cannot be excluded. Second, information on diabetes duration was unavailable in TriNetX. Because diabetes duration is a strong independent predictor of neuropathy, peripheral arterial disease, and foot ulceration, this represents a more consequential limitation than covariate adjustment alone can address; the HbA1c levels and diabetes-related complications used as proxies capture disease severity at a single time point but cannot substitute for cumulative disease exposure, and residual confounding by unmeasured diabetes duration likely remains. Third, the database lacks granular information on podiatry visits, therapeutic footwear, structured ulcer-prevention programs, and vascular screening, all of which meaningfully influence diabetic foot outcomes and were not available for adjustment or description; differential access to these services across treatment groups, consistent with the differences in care intensity noted above, may have contributed to the observed associations. Fourth, the follow-up period was relatively short, reflecting the recent approval of TZP. Additionally, outcome ascertainment relied on routinely collected diagnosis and procedure codes, some of which, such as non-pressure lower-extremity ulcer, cellulitis, and peripheral vascular disease codes, are not specific to diabetic foot disease. Although component-level analyses showed broadly consistent findings, some degree of outcome misclassification cannot be excluded. Although all cohorts were restricted to the same calendar eligibility period, calendar time was not separately incorporated into PSM. Residual temporal confounding may therefore remain because treatment patterns, availability of newer glucose-lowering therapies, and diabetes care practices may have changed during the study period. The median follow-up was 316 days (IQR, 239 days) versus 365 days (IQR, 0 days) for the TZP versus SU cohort, 282 days (IQR, 258 days) versus 365 days (IQR, 0 days) for the TZP versus DPP-4i cohort, 312 days (IQR, 240 days) versus 365 days (IQR, 0 days) for the TZP versus SGLT-2i cohort, and 284 days (IQR, 259 days) versus 365 days (IQR, 0 days) for the TZP versus GLP-1RA cohort. Because diabetic foot ulceration and its sequelae typically develop over several years, a maximum follow-up of 1 year may underestimate the true differences between treatment groups. Longer-term studies are therefore needed to determine whether the observed associations persist, strengthen, or attenuate over time. Fifth, given the observational design, causal inferences cannot be drawn; randomized controlled trials are needed to determine whether TZP directly reduces the risk of diabetic foot complications. Lastly, metformin, although the first-line therapy for T2D worldwide, was not evaluated as a separate comparator in this study. Because most of patients initiating TZP, SU, DPP-4i, SGLT2i, or GLP-1RA in this population were already receiving metformin as background therapy, a cohort of metformin new-users would predominantly capture a distinct, treatment-naïve population early in the disease course rather than one clinically comparable to patients escalating to a second-line or injectable agent. Such a comparison would be confounded by disease stage and duration rather than isolating a drug-class effect. We therefore accounted for metformin use as a baseline covariate in PSM rather than as an independent exposure.

## Conclusion

In summary, this large real-world study shows that TZP was associated with lower rates of diabetic foot complications compared with sulfonylureas, DPP-4is and SGLT2is, with outcomes similar to those observed with GLP-1RA. These observational findings require confirmation in randomized prospective studies.

## Data Availability

The raw data supporting the conclusions of this article will be made available by the authors, without undue reservation.
