# Supplementary material for: Tirzepatide versus other anti-diabetic agents for the prevention of diabetic foot complications in patients with type 2 diabetes
Source: Front Pharmacol. 2026 Sep 24;17:1880132. doi: 10.3389/fphar.2026.1880132 (PMC13645759; doi:10.3389/fphar.2026.1880132)
Supplement: Supplementary file 1 [file DataSheet1.docx]

**Supplemental Table 1.** Demographic, diagnostic, procedural, medication, visit, and laboratory codes utilized in the definition of the cohorts.

| **Category** | **Code** | **Description** |
| --- | --- | --- |
| **T2D** | | |
| Diagnosis | UMLS:ICD10CM:E11 | Type 2 diabetes mellitus |
| **Anti-diabetic medication** | | |
| Medication | NLM:RXNORM:2601723 | Tirzepatide |
| Medication | NLM:ATC:A10BJ | Glucagon-like peptide-1 (GLP-1) analogues |
| Medication | NLM:ATC:A10BH | Dipeptidyl peptidase 4 (DPP-4) inhibitors |
| Medication | NLM:ATC:A10BK | Sodium-glucose co-transporter 2 (SGLT2) inhibitors |
| Medication | NLM:ATC:A10BB | Sulfonylurea |

**Supplemental Table 2.** Definitions of covariates coding used in this study.

| **Code** | **Description** |
| --- | --- |
| AI | Age at Index |
| F | Female |
| M | Male |
| Mar-06 | White |
| May-54 | Black or African American |
| Sep-28 | Asian |
| Jan-31 | Other Race |
| UNK | Unknown Race |
| 9083 | Body mass index |
| 9083 | Body mass index, at least 30 kg/m2 |
| 9037 | Hemoglobin A1c/Hemoglobin.total in Blood |
| 9037 | Hemoglobin A1c/Hemoglobin.total in Blood, at least 9% |
| I10 | Essential (primary) hypertension |
| E78 | Disorders of lipoprotein metabolism and other lipidemias |
| J40-J4A | Chronic lower respiratory diseases |
| I20-I25 | Ischemic heart diseases |
| C00-D49 | Neoplasms |
| N18 | Chronic kidney disease |
| I60-I69 | Cerebrovascular diseases |
| E66 | Overweight and obesity |
| G47.33 | Obstructive sleep apnea |
| I50 | Heart failure |
| E11.2 | Type 2 diabetes mellitus with kidney complications |
| E11.3 | Type 2 diabetes mellitus with ophthalmic complications |
| E11.4 | Type 2 diabetes mellitus with neurological complications |
| E11.5 | Type 2 diabetes mellitus with circulatory complications |
| I48 | Atrial fibrillation and flutter |
| F17 | Nicotine dependence |
| F10 | Alcohol related disorders |
| C10AA | HMG CoA reductase inhibitors |
| C10AB | Fibrates |
| 341248 | Ezetimibe |
| A10BA | Biguanides |
| A10BJ | Glucagon-like peptide-1 analogues |
| A10BK | Sodium-glucose co-transporter 2 inhibitors |
| A10BB | Sulfonylureas |
| A10BG | Thiazolidinediones |
| A10BF | Alpha glucosidase inhibitors |
| A10A | Insulin and analogues |
| CV700 | Diuretics |
| CV100 | Beta blockers/related |
| CV805 | Angiotensin II inhibitor |
| CV800 | Angiotensin-converting enzyme inhibitors |
| CV200 | Calcium channel blockers |
| 1191 | Aspirin |
| 32968 | Clopidogrel |
| 1116632 | Ticagrelor |
| 1656052 | Cangrelor |
| 613391 | Prasugrel |
| 4917 | Nitroglycerin |
| 35829 | Ranolazine |
| 9045 | Albumin [Mass/volume] in Serum, Plasma or Blood |
| 9002 | Cholesterol in LDL [Mass/volume] in Serum or Plasma |
| 9063 | C reactive protein [Mass/volume] in Serum, Plasma or Blood |
| 8001 | Glomerular filtration rate/1.73 sq M.predicted [Volume Rate/Area] in Serum, Plasma or Blood by Creatinine-based formula (MDRD) |

**Supplemental Table 3.** Definitions of outcomes coding used in this study.

| **Code** | **Description** |
| --- | --- |
| **DM foot ulcer or infection** | |
| UMLS:ICD10CM:L97 | Non-pressure chronic ulcer of lower limb, not elsewhere classified |
| UMLS:ICD10CM:E11.621 | Type 2 diabetes mellitus with foot ulcer |
| UMLS:ICD10CM:L89.5 | Pressure ulcer of ankle |
| UMLS:ICD10CM:L89.6 | Pressure ulcer of heel |
| UMLS:ICD10CM:L02.6 | Cutaneous abscess, furuncle and carbuncle of foot |
| UMLS:ICD10CM:L02.415 | Cutaneous abscess of right lower limb |
| UMLS:ICD10CM:L02.416 | Cutaneous abscess of left lower limb |
| UMLS:ICD10CM:L02.425 | Furuncle of right lower limb |
| UMLS:ICD10CM:L02.426 | Furuncle of left lower limb |
| UMLS:ICD10CM:L02.435 | Carbuncle of right lower limb |
| UMLS:ICD10CM:L02.436 | Carbuncle of left lower limb |
| UMLS:ICD10CM:L08 | Other local infections of skin and subcutaneous tissue |
| UMLS:ICD10CM:L04.3 | Acute lymphadenitis of lower limb |
| UMLS:ICD10CM:L03.03 | Cellulitis of toe |
| UMLS:ICD10CM:L03.04 | Acute lymphangitis of toe |
| UMLS:ICD10CM:L03.115 | Cellulitis of right lower limb |
| UMLS:ICD10CM:L03.116 | Cellulitis of left lower limb |
| UMLS:ICD10CM:L03.125 | Acute lymphangitis of right lower limb |
| UMLS:ICD10CM:L03.126 | Acute lymphangitis of left lower limb |
| UMLS:ICD10CM:M86.8 | Other osteomyelitis |
| UMLS:ICD10CM:M86.05 | Acute hematogenous osteomyelitis, femur |
| UMLS:ICD10CM:M86.06 | Acute hematogenous osteomyelitis, tibia and fibula |
| UMLS:ICD10CM:M86.07 | Acute hematogenous osteomyelitis, ankle and foot |
| UMLS:ICD10CM:M86.16 | Other acute osteomyelitis, tibia and fibula |
| UMLS:ICD10CM:M86.17 | Other acute osteomyelitis, ankle and foot |
| UMLS:ICD10CM:M86.25 | Subacute osteomyelitis, femur |
| UMLS:ICD10CM:M86.26 | Subacute osteomyelitis, tibia and fibula |
| UMLS:ICD10CM:M86.27 | Subacute osteomyelitis, ankle and foot |
| UMLS:ICD10CM:M86.35 | Chronic multifocal osteomyelitis, femur |
| UMLS:ICD10CM:M86.36 | Chronic multifocal osteomyelitis, tibia and fibula |
| UMLS:ICD10CM:M86.37 | Chronic multifocal osteomyelitis, ankle and foot |
| UMLS:ICD10CM:M86.45 | Chronic osteomyelitis with draining sinus, femur |
| UMLS:ICD10CM:M86.46 | Chronic osteomyelitis with draining sinus, tibia and fibula |
| UMLS:ICD10CM:M86.47 | Chronic osteomyelitis with draining sinus, ankle and foot |
| UMLS:ICD10CM:M86.55 | Other chronic hematogenous osteomyelitis, femur |
| UMLS:ICD10CM:M86.56 | Other chronic hematogenous osteomyelitis, tibia and fibula |
| UMLS:ICD10CM:M86.57 | Other chronic hematogenous osteomyelitis, ankle and foot |
| UMLS:ICD10CM:M86.65 | Other chronic osteomyelitis, thigh |
| UMLS:ICD10CM:M86.66 | Other chronic osteomyelitis, tibia and fibula |
| UMLS:ICD10CM:M86.67 | Other chronic osteomyelitis, ankle and foot |
| UMLS:ICD10CM:M86.159 | Other acute osteomyelitis, unspecified femur |
| UMLS:ICD10CM:E11.52 | Type 2 diabetes mellitus with diabetic peripheral angiopathy with gangrene |
| UMLS:ICD10CM:I70.25 | Atherosclerosis of native arteries of other extremities with ulceration |
| UMLS:ICD10CM:I70.269 | Atherosclerosis of native arteries of extremities with gangrene, unspecified extremity |
| UMLS:ICD10CM:I96 | Gangrene, not elsewhere classified |
| UMLS:ICD10CM:A48.0 | Gas gangrene |
| **Critical limb ischemia (Peripheral arterial occlusive disease, gangrene)** | |
| UMLS:ICD10CM:I70.22 | Atherosclerosis of native arteries of extremities with rest pain |
| UMLS:ICD10CM:I73.9 | Peripheral vascular disease, unspecified |
| UMLS:ICD10CM:I70.25 | Atherosclerosis of native arteries of other extremities with ulceration |
| UMLS:ICD10CM:I70.26 | Atherosclerosis of native arteries of extremities with gangrene |
| **Receive procedure (revascularization and amputation)** | |
| UMLS:CPT:1014580 | Amputation, thigh, through femur, any level |
| UMLS:CPT:1014590 | Amputation, leg, through tibia and fibula |
| UMLS:CPT:27590 | Amputation, thigh, through femur, any level |
| UMLS:CPT:27888 | Amputation, ankle, through malleoli of tibia and fibula (eg, Syme, Pirogoff type procedures), with plastic closure and resection of nerves |
| UMLS:CPT:27889 | Ankle disarticulation |
| UMLS:CPT:1005525 | Amputation, foot |
| UMLS:CPT:1005529 | Amputation, toe |
| UMLS:CPT:28810 | Amputation, metatarsal, with toe, single |
| UMLS:CPT:28122 | Partial excision (craterization, saucerization, sequestrectomy, or diaphysectomy) bone (eg, osteomyelitis or bossing); tarsal or metatarsal bone, except talus or calcaneus |
| UMLS:CPT:27598 | Disarticulation at knee |
| UMLS:CPT:1020565 | Revascularization, endovascular, open or percutaneous, iliac artery, unilateral, initial vessel |
| UMLS:CPT:1020566 | Revascularization, endovascular, open or percutaneous, iliac artery, each additional ipsilateral iliac vessel |
| UMLS:CPT:1020567 | Revascularization, endovascular, open or percutaneous, femoral, popliteal artery(s), unilateral |
| UMLS:CPT:1020568 | Revascularization, endovascular, open or percutaneous, tibial, peroneal artery, unilateral, initial vessel |
| UMLS:CPT:1020569 | Revascularization, endovascular, open or percutaneous, tibial/peroneal artery, unilateral, each additional vessel |
| **Non-pressure ulcer** | |
| UMLS:ICD10CM:L97 | Non-pressure chronic ulcer of lower limb, not elsewhere classified |
| UMLS:ICD10CM:E11.621 | Type 2 diabetes mellitus with foot ulcer |
| Pressure ulcer |  |
| UMLS:ICD10CM:L89.5 | Pressure ulcer of ankle |
| UMLS:ICD10CM:L89.6 | Pressure ulcer of heel |
| **Cellulitis** | |
| UMLS:ICD10CM:L03.03 | Cellulitis of toe |
| UMLS:ICD10CM:L03.115 | Cellulitis of right lower limb |
| UMLS:ICD10CM:L03.116 | Cellulitis of left lower limb |
| UMLS:ICD10CM:L03.04 | Acute lymphangitis of toe |
| UMLS:ICD10CM:L03.125 | Acute lymphangitis of right lower limb |
| UMLS:ICD10CM:L03.126 | Acute lymphangitis of left lower limb |
| **Abscess** | |
| UMLS:ICD10CM:L02.6 | Cutaneous abscess, furuncle and carbuncle of foot |
| UMLS:ICD10CM:L02.415 | Cutaneous abscess of right lower limb |
| UMLS:ICD10CM:L02.416 | Cutaneous abscess of left lower limb |
| UMLS:ICD10CM:L02.425 | Furuncle of right lower limb |
| UMLS:ICD10CM:L02.426 | Furuncle of left lower limb |
| UMLS:ICD10CM:L02.435 | Carbuncle of right lower limb |
| UMLS:ICD10CM:L02.436 | Carbuncle of left lower limb |
| **Osteomyelitis** | |
| UMLS:ICD10CM:M86.8 | Other osteomyelitis |
| UMLS:ICD10CM:M86.05 | Acute hematogenous osteomyelitis, femur |
| UMLS:ICD10CM:M86.06 | Acute hematogenous osteomyelitis, tibia and fibula |
| UMLS:ICD10CM:M86.07 | Acute hematogenous osteomyelitis, ankle and foot |
| UMLS:ICD10CM:M86.16 | Other acute osteomyelitis, tibia and fibula |
| UMLS:ICD10CM:M86.17 | Other acute osteomyelitis, ankle and foot |
| UMLS:ICD10CM:M86.25 | Subacute osteomyelitis, femur |
| UMLS:ICD10CM:M86.26 | Subacute osteomyelitis, tibia and fibula |
| UMLS:ICD10CM:M86.27 | Subacute osteomyelitis, ankle and foot |
| UMLS:ICD10CM:M86.35 | Chronic multifocal osteomyelitis, femur |
| UMLS:ICD10CM:M86.36 | Chronic multifocal osteomyelitis, tibia and fibula |
| UMLS:ICD10CM:M86.37 | Chronic multifocal osteomyelitis, ankle and foot |
| UMLS:ICD10CM:M86.45 | Chronic osteomyelitis with draining sinus, femur |
| UMLS:ICD10CM:M86.46 | Chronic osteomyelitis with draining sinus, tibia and fibula |
| UMLS:ICD10CM:M86.47 | Chronic osteomyelitis with draining sinus, ankle and foot |
| UMLS:ICD10CM:M86.55 | Other chronic hematogenous osteomyelitis, femur |
| UMLS:ICD10CM:M86.56 | Other chronic hematogenous osteomyelitis, tibia and fibula |
| UMLS:ICD10CM:M86.57 | Other chronic hematogenous osteomyelitis, ankle and foot |
| UMLS:ICD10CM:M86.65 | Other chronic osteomyelitis, thigh |
| UMLS:ICD10CM:M86.66 | Other chronic osteomyelitis, tibia and fibula |
| UMLS:ICD10CM:M86.67 | Other chronic osteomyelitis, ankle and foot |
| UMLS:ICD10CM:M86.159 | Other acute osteomyelitis, unspecified femur |
| **PAOD/CLI** | |
| UMLS:ICD10CM:I70.22 | Atherosclerosis of native arteries of extremities with rest pain |
| UMLS:ICD10CM:I73.9 | Peripheral vascular disease, unspecified |
| UMLS:ICD10CM:I70.25 | Atherosclerosis of native arteries of other extremities with ulceration |
| UMLS:ICD10CM:I70.26 | Atherosclerosis of native arteries of extremities with gangrene |
| **Gangrene** | |
| UMLS:ICD10CM:E11.52 | Type 2 diabetes mellitus with diabetic peripheral angiopathy with gangrene |
| UMLS:ICD10CM:I70.269 | Atherosclerosis of native arteries of extremities with gangrene, unspecified extremity |
| UMLS:ICD10CM:I96 | Gangrene, not elsewhere classified |
| UMLS:ICD10CM:A48.0 | Gas gangrene |
| UMLS:ICD10CM:I70.26 | Atherosclerosis of native arteries of extremities with gangrene |
| **Revascularization** | |
| UMLS:CPT:1020565 | Revascularization, endovascular, open or percutaneous, iliac artery, unilateral, initial vessel |
| UMLS:CPT:1020566 | Revascularization, endovascular, open or percutaneous, iliac artery, each additional ipsilateral iliac vessel |
| UMLS:CPT:1020567 | Revascularization, endovascular, open or percutaneous, femoral, popliteal artery(s), unilateral |
| UMLS:CPT:1020568 | Revascularization, endovascular, open or percutaneous, tibial, peroneal artery, unilateral, initial vessel |
| UMLS:CPT:1020569 | Revascularization, endovascular, open or percutaneous, tibial/peroneal artery, unilateral, each additional vessel |
| **Minor amputation** | |
| UMLS:CPT:1005525 | Amputation, foot |
| UMLS:CPT:1005529 | Amputation, toe |
| UMLS:CPT:28810 | Amputation, metatarsal, with toe, single |
| **Major amputation** | |
| UMLS:CPT:1014580 | Amputation, thigh, through femur, any level |
| UMLS:CPT:1014590 | Amputation, leg, through tibia and fibula |
| UMLS:CPT:27590 | Amputation, thigh, through femur, any level |
| UMLS:CPT:27888 | Amputation, ankle, through malleoli of tibia and fibula (eg, Syme, Pirogoff type procedures), with plastic closure and resection of nerves |
| UMLS:CPT:27889 | Ankle disarticulation |
| UMLS:CPT:27598 | Disarticulation at knee |

**Supplemental Table 4**. Pre-match baseline characteristics of TZP and other antidiabetic medication cohorts.

| Variables | TZP | SU | SMD | TZP | DPP-4i | SMD | TZP | SGLT-2i | SMD | TZP | GLP-1RA | SMD |
| --- | --- | --- | --- | --- | --- | --- | --- | --- | --- | --- | --- | --- |
| Sample size in each cohort (n) | 184,681 | 705,905 |  | 207,010 | 517,758 |  | 161,671 | 794,769 |  | 97,458 | 766,620 |  |
| Age, years |  |  |  |  |  |  |  |  |  |  |  |  |
| Mean (SD) | 56.4 (12.5) | 62 (13.1) | 0.444 | 56.6 (12.4) | 63.7 (13.1) | 0.556 | 56.3 (12.6) | 63.1 (12.7) | 0.542 | 57.5 (12.6) | 58.4 (12.8) | 0.07 |
| Sex, n (%) |  |  |  |  |  |  |  |  |  |  |  |  |
| Female | 104,852 (59.6) | 320,919 (46.8) | 0.259 | 122,391 (58.7) | 253,079 (48.7) | 0.201 | 103,690 (61.7) | 347,458 (42.5) | 0.392 | 53,874 (55) | 411,678 (53.5) | 0.03 |
| Male | 67,266 (38.2) | 353,608 (51.5) | 0.27 | 82,091 (39.4) | 258,450 (49.7) | 0.21 | 60,928 (36.3) | 453,545 (55.5) | 0.394 | 42,325 (43.2) | 341,260 (44.3) | 0.023 |
| Race, n (%) |  |  |  |  |  |  |  |  |  |  |  |  |
| White | 118,423 (67.3) | 395,723 (57.7) | 0.2 | 141,211 (67.7) | 253,232 (48.7) | 0.392 | 113,144 (67.4) | 465,089 (56.9) | 0.216 | 67,459 (68.8) | 475,937 (61.8) | 0.147 |
| Black or African American | 29,394 (16.7) | 111,069 (16.2) | 0.014 | 35,421 (17) | 78,453 (15.1) | 0.051 | 29,301 (17.4) | 135,133 (16.5) | 0.024 | 15,751 (16.1) | 144,393 (18.8) | 0.071 |
| Asian | 5,897 (3.4) | 48,065 (7) | 0.165 | 6,625 (3.2) | 52,992 (10.2) | 0.284 | 5,034 (3) | 58,785 (7.2) | 0.192 | 3,327 (3.4) | 34,565 (4.5) | 0.056 |
| Other Race | 7,020 (4) | 27,506 (4) | 0.001 | 7,966 (3.8) | 18,759 (3.6) | 0.011 | 6,412 (3.8) | 35,004 (4.3) | 0.024 | 3,673 (3.7) | 34,541 (4.5) | 0.037 |
| Unknown Race | 12,805 (7.3) | 93,595 (13.6) | 0.209 | 14,652 (7) | 109,011 (21) | 0.411 | 11,994 (7.1) | 111,837 (13.7) | 0.216 | 6,686 (6.8) | 68,392 (8.9) | 0.077 |
| Body mass index, kg/m² |  |  |  |  |  |  |  |  |  |  |  |  |
| ≥ 30, n (%) | 132,786 (71.9) | 280,950 (39.8) | 0.685 | 149,254 (72.1) | 164,129 (31.7) | 0.884 | 117,050 (72.4) | 348,904 (43.9) | 0.604 | 65,882 (67.6) | 453,839 (59.2) | 0.175 |
| Hemoglobin A1c, % |  |  |  |  |  |  |  |  |  |  |  |  |
| ≥ 9, n (%) | 23,270 (12.6) | 141,887 (20.1) | 0.202 | 31,052 (15.0) | 96,303 (18.6) | 0.099 | 21,341 (13.2) | 156,569 (19.7) | 0.177 | 13,936 (14.3) | 179,389 (23.4) | 0.232 |
| Comorbidities, n (%) |  |  |  |  |  |  |  |  |  |  |  |  |
| Hypertension | 105,021 (59.7) | 393,427 (57.3) | 0.048 | 125,651 (60.2) | 301,118 (58) | 0.047 | 98,548 (58.7) | 503,011 (61.6) | 0.059 | 56,582 (57.7) | 460,951 (59.9) | 0.044 |
| Dyslipidemia | 104,166 (59.2) | 359,272 (52.4) | 0.138 | 124,860 (59.9) | 282,649 (54.4) | 0.111 | 96,343 (57.3) | 493,344 (60.4) | 0.062 | 55,262 (56.4) | 437,848 (56.9) | 0.01 |
| Chronic lower respiratory diseases | 29,757 (16.9) | 84,556 (12.3) | 0.13 | 34,636 (16.6) | 65,061 (12.5) | 0.116 | 27,935 (16.6) | 127,834 (15.6) | 0.027 | 15,435 (15.7) | 114,570 (14.9) | 0.024 |
| Ischemic heart disease | 23,401 (13.3) | 110,144 (16.1) | 0.078 | 27,752 (13.3) | 93,700 (18) | 0.13 | 18,685 (11.1) | 215,352 (26.4) | 0.398 | 13,217 (13.5) | 114,763 (14.9) | 0.041 |
| Neoplasms | 32,218 (18.3) | 96,491 (14.1) | 0.116 | 37,858 (18.2) | 80,204 (15.4) | 0.073 | 30,630 (18.2) | 132,123 (16.2) | 0.055 | 16,746 (17.1) | 119,204 (15.5) | 0.043 |
| Chronic kidney disease | 17,811 (10.1) | 80,379 (11.7) | 0.051 | 21,550 (10.3) | 81,666 (15.7) | 0.16 | 14,840 (8.8) | 152,722 (18.7) | 0.289 | 10,113 (10.3) | 91,865 (11.9) | 0.051 |
| Cerebrovascular diseases | 7,059 (4) | 44,784 (6.5) | 0.113 | 8,435 (4) | 41,947 (8.1) | 0.169 | 6,226 (3.7) | 67,426 (8.3) | 0.193 | 4,205 (4.3) | 39,471 (5.1) | 0.04 |
| Overweight and obesity | 82,708 (47) | 130,571 (19) | 0.623 | 96,788 (46.4) | 90,244 (17.4) | 0.656 | 78,854 (46.9) | 204,882 (25.1) | 0.468 | 38,758 (39.5) | 258,903 (33.6) | 0.123 |
| Obstructive sleep apnea | 38,808 (22.1) | 54,291 (7.9) | 0.404 | 45,535 (21.8) | 38,664 (7.4) | 0.416 | 35,559 (21.2) | 106,763 (13.1) | 0.216 | 18,821 (19.2) | 118,681 (15.4) | 0.1 |
| Heart failure | 12,680 (7.2) | 50,901 (7.4) | 0.008 | 15,111 (7.2) | 44,877 (8.6) | 0.051 | 8,180 (4.9) | 159,251 (19.5) | 0.459 | 7,145 (7.3) | 59,692 (7.8) | 0.018 |
| Atrial fibrillation and flutter | 10,218 (5.8) | 45,432 (6.6) | 0.034 | 12,155 (5.8) | 38,406 (7.4) | 0.063 | 8,350 (5) | 100,026 (12.2) | 0.262 | 6,175 (6.3) | 46,410 (6) | 0.011 |
| Nicotine dependence | 11,446 (6.5) | 50,069 (7.3) | 0.031 | 13,494 (6.5) | 32,922 (6.3) | 0.005 | 10,618 (6.3) | 69,535 (8.5) | 0.084 | 6,577 (6.7) | 57,519 (7.5) | 0.03 |
| Alcohol related disorders | 2,430 (1.4) | 10,879 (1.6) | 0.017 | 2,822 (1.4) | 6,880 (1.3) | 0.003 | 2,214 (1.3) | 15,781 (1.9) | 0.049 | 1,479 (1.5) | 10,381 (1.3) | 0.014 |
| Type 2 diabetes complications |  |  |  |  |  |  |  |  |  |  |  |  |
| Kidney complications | 17,607 (10) | 71,631 (10.4) | 0.014 | 21,827 (10.5) | 71,350 (13.7) | 0.1 | 14,414 (8.6) | 141,345 (17.3) | 0.262 | 9,170 (9.4) | 90,653 (11.8) | 0.079 |
| Ophthalmic complications | 7,359 (4.2) | 23,634 (3.4) | 0.039 | 9,371 (4.5) | 22,635 (4.4) | 0.007 | 6,072 (3.6) | 43,260 (5.3) | 0.082 | 3,314 (3.4) | 37,073 (4.8) | 0.072 |
| Neurological complications | 17,325 (9.8) | 57,224 (8.3) | 0.053 | 21,783 (10.4) | 48,350 (9.3) | 0.038 | 15,088 (9) | 97,254 (11.9) | 0.096 | 8,395 (8.6) | 84,873 (11) | 0.083 |
| Circulatory complications | 9,800 (5.6) | 26,791 (3.9) | 0.079 | 12,072 (5.8) | 22,128 (4.3) | 0.07 | 8,640 (5.1) | 54,500 (6.7) | 0.065 | 5,229 (5.3) | 39,064 (5.1) | 0.012 |
| Medication, n (%) |  |  |  |  |  |  |  |  |  |  |  |  |
| HMG CoA reductase inhibitors | 79,693 (45.3) | 247,692 (36.1) | 0.188 | 97,516 (46.8) | 220,865 (42.5) | 0.086 | 72,404 (43.1) | 420,142 (51.4) | 0.167 | 41,573 (42.4) | 357,221 (46.4) | 0.08 |
| Fibrates | 5,391 (3.1) | 20,330 (3) | 0.006 | 6,887 (3.3) | 17,916 (3.4) | 0.008 | 4,481 (2.7) | 28,820 (3.5) | 0.05 | 2,871 (2.9) | 26,588 (3.5) | 0.03 |
| Ezetimibe | 6,384 (3.6) | 12,906 (1.9) | 0.107 | 7,660 (3.7) | 13,830 (2.7) | 0.058 | 5,033 (3) | 30,681 (3.8) | 0.042 | 3,186 (3.3) | 22,127 (2.9) | 0.022 |
| Diuretics | 50,322 (28.6) | 154,017 (22.4) | 0.142 | 60,704 (29.1) | 131,612 (25.3) | 0.085 | 46,162 (27.5) | 276,412 (33.8) | 0.138 | 26,494 (27) | 218,300 (28.4) | 0.03 |
| Beta blockers related | 44,583 (25.3) | 165,697 (24.1) | 0.028 | 53,583 (25.7) | 143,219 (27.6) | 0.042 | 39,539 (23.5) | 286,415 (35.1) | 0.255 | 24,360 (24.8) | 202,505 (26.3) | 0.033 |
| Angiotensin II inhibitors | 41,868 (23.8) | 101,379 (14.8) | 0.23 | 50,217 (24.1) | 99,725 (19.2) | 0.119 | 37,115 (22.1) | 207,401 (25.4) | 0.077 | 21,860 (22.3) | 160,178 (20.8) | 0.036 |
| Angiotensin-converting enzyme inhibitors | 32,084 (18.2) | 129,931 (18.9) | 0.018 | 40,286 (19.3) | 100,550 (19.4) | 0.001 | 30,586 (18.2) | 182,604 (22.4) | 0.103 | 17,626 (18) | 174,350 (22.6) | 0.116 |
| Calcium channel blocker | 33,298 (18.9) | 120,899 (17.6) | 0.034 | 40,578 (19.5) | 117,065 (22.5) | 0.076 | 31,714 (18.9) | 204,592 (25) | 0.149 | 18,826 (19.2) | 154,953 (20.1) | 0.023 |
| Aspirin | 18,415 (10.5) | 105,353 (15.4) | 0.146 | 22,277 (10.7) | 94,949 (18.3) | 0.217 | 15,711 (9.4) | 178,413 (21.8) | 0.349 | 10,374 (10.6) | 110,854 (14.4) | 0.116 |
| Clopidogrel | 5,265 (3) | 31,413 (4.6) | 0.083 | 6,544 (3.1) | 30,177 (5.8) | 0.129 | 4,155 (2.5) | 64,086 (7.8) | 0.245 | 3,240 (3.3) | 34,504 (4.5) | 0.061 |
| Ticagrelor | 626 (0.4) | 3,735 (0.5) | 0.028 | 790 (0.4) | 3,488 (0.7) | 0.04 | 454 (0.3) | 11,427 (1.4) | 0.124 | 362 (0.4) | 4,918 (0.6) | 0.038 |
| Cangrelor | 20 (0) | 106 (0) | 0.004 | 24 (0) | 75 (0) | 0.003 | 11 (0) | 535 (0.1) | 0.031 | 15 (0) | 138 (0) | 0.002 |
| Prasugrel | 368 (0.2) | 1,553 (0.2) | 0.004 | 455 (0.2) | 1,579 (0.3) | 0.017 | 268 (0.2) | 4,037 (0.5) | 0.059 | 226 (0.2) | 2,250 (0.3) | 0.012 |
| Nitroglycerin | 7,155 (4.1) | 40,898 (6) | 0.087 | 8,519 (4.1) | 35,704 (6.9) | 0.123 | 5,676 (3.4) | 79,830 (9.8) | 0.26 | 4,045 (4.1) | 39,197 (5.1) | 0.046 |
| Ranolazine | 640 (0.4) | 2,021 (0.3) | 0.012 | 769 (0.4) | 1,916 (0.4) | 0 | 413 (0.2) | 5,860 (0.7) | 0.068 | 358 (0.4) | 3,128 (0.4) | 0.007 |
| Biguanides | 67,737 (38.5) | 247,462 (36.1) | 0.051 | 83,369 (40) | 200,492 (38.6) | 0.028 | 63,866 (38) | 332,382 (40.7) | 0.055 | 37,728 (38.5) | 346,994 (45.1) | 0.134 |
| Thiazolidinediones | 3,746 (2.1) | 19,041 (2.8) | 0.042 | 5,610 (2.7) | 19,094 (3.7) | 0.056 | 3,669 (2.2) | 28,734 (3.5) | 0.08 | 2,492 (2.5) | 28,020 (3.6) | 0.063 |
| Alpha glucosidase inhibitors | 174 (0.1) | 2,562 (0.4) | 0.057 | 242 (0.1) | 3,862 (0.7) | 0.096 | 187 (0.1) | 4,243 (0.5) | 0.073 | 109 (0.1) | 2,332 (0.3) | 0.042 |
| Insulins and analogues | 40,142 (22.8) | 161,294 (23.5) | 0.016 | 50,447 (24.2) | 139,660 (26.9) | 0.062 | 34,264 (20.4) | 258,782 (31.7) | 0.259 | 19,051 (19.4) | 217,057 (28.2) | 0.207 |
| Sulfonylureas | - | - | - | 20,391 (9.8) | 101,539 (19.5) | 0.279 | 14,904 (8.9) | 141,271 (17.3) | 0.252 | 41,573 (42.4) | 357,221 (46.4) | 0.08 |
| Dipeptidyl peptidase 4 inhibitors | 19,091 (9.7) | 97,793 (19.7) | 0.287 | - | - | - | 37,223 (18.8) | 44,571 (9) | 0.287 | 5,945 (6.1) | 86,322 (11.2) | 0.184 |
| Sodium-glucose co-transporter 2 inhibitors | 30,797 (17.5) | 52,773 (7.7) | 0.299 | 38,760 (18.6) | 46,812 (9) | 0.28 | - | - | - | 10,018 (10.2) | 136,374 (17.7) | 0.217 |
| Glucagon-like peptide-1 analogues | 77,165 (43.9) | 41,449 (6) | 0.972 | 94,446 (45.3) | 25,601 (4.9) | 1.052 | 70,938 (42.2) | 116,295 (14.2) | 0.654 | - | - | - |
| Lab data |  |  |  |  |  |  |  |  |  |  |  |  |
| LDL-C, mg/dL | 91 ± 37.3 | 91.6 ± 38.9 | 0.016 | 90.2 ± 37.4 | 90 ± 37.7 | 0.006 | 92.6 ± 37.3 | 85.2 ± 37.5 | 0.198 | 92.5 ± 38.1 | 90.5 ± 38.0 | 0.053 |
| Albumin, g/dL | 4.1 ± 0.4 | 4.0 ± 0.6 | 0.313 | 4.1 ± 0.4 | 3.9 ± 0.6 | 0.398 | 4.1 ± 0.4 | 4.0 ± 0.6 | 0.298 | 4.1 ± 0.4 | 4.1 ± 0.5 | 0.068 |
| C-reactive protein, mg/L | 23.2 ± 46.6 | 40.7 ± 65.1 | 0.308 | 23.8 ± 47.3 | 38.8 ± 63.1 | 0.268 | 23.1 ± 46.1 | 36.1 ± 60.3 | 0.243 | 25.9 ± 49.7 | 29.9 ± 54.4 | 0.078 |
| eGFR, mL/min/1.73 m² | 81.3 ± 26.5 | 77.8 ± 31.7 | 0.119 | 81.4 ± 26.7 | 73.5 ± 32.9 | 0.263 | 82.1 ± 26.4 | 74 ± 29.6 | 0.289 | 80.4 ± 27.1 | 79.5 ± 28.6 | 0.034 |

DPP-4i, dipeptidyl peptidase 4 inhibitor; eGFR, estimated Glomerular filtration rate; GLP-1RA, glucagon-like peptide-1 receptor agonist; LDL-C, low-density lipoprotein cholesterol; SGLT2i, sodium-glucose cotransporter 2 inhibitor; SMD, standardized mean difference SU, sulfonylurea; TZP, Tirzepatide

**Supplemental Table 5**. One-year absolute risk reduction and number needed to treat for diabetic foot complications.

| Comparison | One-year risk, TZP | One-year risk, Comparator | Absolute risk reduction | NNT (95% CI) |
| --- | --- | --- | --- | --- |
| TZP vs SU | 1.65% | 2.77% | 1.12% | 89 (81 – 98) |
| TZP vs DPP-4i | 1.63% | 2.82% | 1.19% | 84 (77 – 93) |
| TZP vs SGLT2i | 1.65% | 2.40% | 0.75% | 133 (118 – 154) |
| TZP vs GLP-1RA | 1.71% | 2.34% | 0.63% | 158 (132 – 197) |

DPP-4i, dipeptidyl peptidase 4 inhibitor; GLP-1RA, glucagon-like peptide-1 receptor agonist; SGLT2i, sodium-glucose cotransporter 2 inhibitor; SU, sulfonylurea; TZP, Tirzepatide

**Supplemental Table 6**. Component-level analyses of diabetic foot complications comparing tirzepatide with other glucose-lowering therapies.

| Component outcome | Events (n); HR (95% CI) | | | |
| --- | --- | --- | --- | --- |
|  | TZP vs. SU | TZP vs. DPP-4i | TZP vs. SGLT-2i | TZP vs. GLP-1RA |
| Non-pressure ulcer | 1,206 vs. 1,949; 0.77 (0.72,0.83) | 1,334 vs. 1,977; 0.85 (0.79,0.91) | 1,479 vs. 2,074; 0.84 (0.78,0.90) | 1,145 vs. 1,370; 1.02 (0.94,1.10) |
| Pressure ulcer | 112 vs. 207; 0.67 (0.53,0.84) | 109 vs. 324; 0.42 (0.34,0.52) | 124 vs. 215; 0.68 (0.54,0.84) | 101 vs. 143; 0.86 (0.67,1.11) |
| Cellulitis | 1,450 vs. 2,358; 0.78 (0.73,0.84) | 1,598 vs. 2,284; 0.90 (0.85,0.96) | 1,907 vs. 2,583; 0.88 (0.83,0.93) | 1,418 vs. 1,803; 0.97 (0.91,1.04) |
| Abscess | 321 vs. 685; 0.58 (0.51,0.67) | 406 vs. 674; 0.75 (0.67,0.85) | 416 vs. 687; 0.71 (0.63,0.80) | 316 vs. 419; 0.92 (0.80,1.07) |
| Osteomyelitis | 330 vs. 599; 0.68 (0.59,0.78) | 405 vs. 656; 0.76 (0.67,0.86) | 374 vs. 649; 0.67 (0.59,0.76) | 317 vs. 414; 0.93 (0.80,1.08) |
| PAOD/CLI | 1,392 vs. 2,094; 0.83 (0.78,0.89) | 1,530 vs. 2,458; 0.78 (0.73,0.83) | 1,706 vs. 2,451; 0.82 (0.77,0.87) | 1,333 vs. 1,633; 0.99 (0.93,1.07) |
| Gangrene | 280 vs. 487; 0.70 (0.61,0.81) | 316 vs. 625; 0.62 (0.54,0.71) | 321 vs. 552; 0.68 (0.59,0.78) | 276 vs. 367; 0.91 (0.78,1.07) |
| Revascularization | 111 vs. 263; 0.51 (0.40,0.63) | 131 vs. 350; 0.45 (0.37,0.55) | 120 vs. 325; 0.42 (0.34,0.52) | 104 vs. 179; 0.69 (0.54,0.88) |
| Minor amputation | 150 vs. 273; 0.67 (0.55,0.81) | 151 vs. 288; 0.64 (0.52,0.78) | 142 vs. 323; 0.50 (0.41,0.61) | 131 vs. 183; 0.86 (0.69,1.08) |
| Major amputation | 56 vs. 96; 0.71 (0.51,0.99) | 60 vs. 137; 0.53 (0.39,0.72) | 53 vs. 113; 0.54 (0.39,0.75) | 48 vs. 72; 0.81 (0.56,1.17) |

CI, confidence interval; CLI, critical limb ischemia; DPP-4i, dipeptidyl peptidase-4 inhibitor; GLP-1RA, glucagon-like peptide-1 receptor agonist; HR, hazard ratio; PAOD, peripheral arterial occlusive disease; SGLT2i, sodium-glucose cotransporter-2 inhibitor; SU, sulfonylurea; TZP, tirzepatide

**Supplemental Table 7**. Sensitivity analysis excluding concurrent use of SGLT2 inhibitors or GLP-1 receptor agonists.

| Comparator | Events (n) | Incidence rate  per 100 PYs | Events (n) | Incidence rate  per 100 PYs | HR (95% CI) | *P* value | E-value  (95% LCL) |
| --- | --- | --- | --- | --- | --- | --- | --- |
| Primary outcome: diabetic foot complication |  |  |  |  |  |  |  |
| Tirzepatide (n = 105,604) vs. SU (n = 105,604) | 1,775 | 1.7 | 3,015 | 2.9 | 0.76 (0.72,0.81) | < 0.0001 | 1.96 (1.77) |
| Tirzepatide (n = 99,353) vs. DPP-4i (n = 99,353) | 1,727 | 1.7 | 2,719 | 2.7 | 0.83 (0.78,0.88) | < 0.0001 | 1.70 (1.53) |
| Secondary outcomes: diabetic foot complication without major adverse limb events | | | | | | | |
| Tirzepatide (n = 105,604) vs. SU (n = 105,604) | 1,705 | 1.6 | 2,823 | 2.7 | 0.79 (0.75,0.84) | < 0.0001 | 1.85 (1.67) |
| Tirzepatide (n = 99,353) vs. DPP-4i (n = 99,353) | 1,663 | 1.7 | 2,495 | 2.5 | 0.87 (0.82,0.93) | < 0.0001 | 1.56 (1.36) |
| Secondary outcomes: diabetic foot complication with major adverse limb events | | | | | | | |
| Tirzepatide (n = 105,604) vs. SU (n = 105,604) | 849 | 0.8 | 1,352 | 1.3 | 0.80 (0.73,0.87) | < 0.0001 | 1.81 (1.56) |
| Tirzepatide (n = 99,353) vs. DPP-4i (n = 99,353) | 846 | 0.9 | 1,325 | 1.3 | 0.81 (0.74,0.88) | < 0.0001 | 1.77 (1.53) |

**Supplemental Table 8**. STROBE and RECORD checklist for the reporting of observational studies using routinely collected health data.

| **Section** | **Item** | **STROBE item** | **Location in revised manuscript** | **RECORD extension** | **Location in revised manuscript** |
| --- | --- | --- | --- | --- | --- |
| **Title and abstract** | 1 | (a) Indicate the study design with a commonly used term in the title or abstract. (b) Provide an informative and balanced abstract summary of what was done and found. | Title; Abstract (Background, Methods, Results, Conclusions) | RECORD 1.1: Specify the type of routinely collected data and, where possible, the database name. RECORD 1.2: Report the geographic region and study timeframe, if applicable. RECORD 1.3: State database linkage if performed. | Abstract—Methods: retrospective cohort using the global TriNetX electronic health record network, Jan 2022–Jul 2025. RECORD 1.3: Not applicable; no external database linkage. |
| **Introduction** | 2 | Explain the scientific background and rationale for the investigation. | Introduction, paragraphs 1–2 |  |  |
| Introduction | 3 | State specific objectives, including any prespecified hypotheses. | Introduction, final paragraph |  |  |
| **Methods** | 4 | Present key elements of the study design early in the paper. | Methods—Data Source; Study Design |  |  |
| Methods | 5 | Describe the setting, locations, and relevant dates, including recruitment/exposure, follow-up, and data collection periods. | Methods—Data Source; Study Design; Outcomes | No additional RECORD item beyond STROBE; describe the content/role of the routine data source and its source population. | Methods—Data Source: global federated EHR network; Study Design: Jan 1, 2022–Jul 31, 2025; Outcomes: follow-up to 1 year. |
| Methods | 6 | Give eligibility criteria and sources/methods of participant selection and follow-up. | Methods—Study Design; Figure 1; Supplemental Table 1 | RECORD 6.1: Describe in detail the codes/algorithms used to select the study population. RECORD 6.2: Reference validation studies of selection codes or algorithms, or report validation performed for the study. RECORD 6.3: If databases were linked, consider a linkage flow diagram. | 6.1: Methods—Study Design; Figure 1; Supplemental Table 1.  6.2: No study-specific validation of the cohort selection codes or algorithms was performed. 6.3: Not applicable; no external database linkage. |
| Methods | 7 | Clearly define outcomes, exposures, predictors, confounders, and effect modifiers; give diagnostic criteria when applicable. | Methods—Study Design; Covariates and Propensity Score Matching; Outcomes | RECORD 7.1: Provide a complete list of codes and algorithms used to classify exposures, outcomes, confounders, and effect modifiers. | Supplemental Tables 1–3 (exposure/eligibility, covariates, and outcome coding); Supplemental Table 6 for component-level outcomes. |
| Methods | 8 | For each variable, give data sources and details of measurement/assessment; describe comparability across groups. | Methods—Data Source; Covariates and Propensity Score Matching; Outcomes; Supplemental Tables 1–3 |  |  |
| Methods | 9 | Describe efforts to address potential sources of bias. | Methods—new-user active comparisons, PSM, incident-outcome exclusions, E-values; Discussion—Limitations |  |  |
| Methods | 10 | Explain how the study size was arrived at. | Methods—Study Design; Results—Patient selection; Figure 1 | No additional RECORD item. | All eligible patients meeting cohort definitions in TriNetX were considered; matched sample sizes are shown in Figure 1 and Results. |
| Methods | 11 | Explain how quantitative variables were handled; describe any groupings and why they were chosen. | Methods—Covariates and Propensity Score Matching; Statistical analysis; subgroup definitions |  |  |
| Methods | 12 | Describe statistical methods, confounding control, subgroup/interaction analyses, missing-data handling, loss to follow-up, matching, and sensitivity analyses as applicable. | Methods—Covariates and Propensity Score Matching; Outcomes; Statistical analysis | RECORD 12.1: Describe investigators’ access to the database used to create the study population. RECORD 12.2: Provide information on data-cleaning methods. RECORD 12.3: State whether person-, institution-, or other-level linkage across databases was performed and describe linkage methods/quality if applicable. | 12.1: Investigators accessed de-identified, aggregate data through the TriNetX analytics platform and did not have access to directly identifiable patient-level data. 12.2: No additional investigator-level data cleaning was performed beyond the data harmonization and standardization implemented within the TriNetX platform.  12.3: Not applicable; no external database linkage. |
| **Results** | 13 | Report numbers of individuals at each stage of the study and reasons for non-participation/exclusion; consider a flow diagram. | Results—Patient selection; Figure 1 | RECORD 13.1: Describe in detail selection of included persons, including filtering based on data quality, availability, and linkage. | Results—Patient selection; Figure 1. No linkage-based filtering was performed. |
| Results | 14 | Give participant characteristics, exposures/confounders, and indicate missing data for variables of interest; summarize follow-up time. | Table 1; Supplemental Table 4; Discussion—Limitations (median follow-up by comparison) | No additional RECORD item. | Table 1/Supplemental Table 4; median follow-up is reported in Discussion. Missing data were not imputed; analyses used available data recorded within TriNetX. |
| Results | 15 | Report numbers of outcome events or summary measures over time. | Results—Primary and Secondary outcomes; Table 2; Supplemental Table 6 |  |  |
| Results | 16 | Give unadjusted and adjusted estimates with precision; state which confounders were adjusted for and why; report category boundaries and translate relative risk into absolute risk when relevant. | Methods—PSM covariates; Results; Table 2; Supplemental Table 5 |  |  |
| Results | 17 | Report other analyses performed, including subgroup and sensitivity analyses. | Figure 3; E-value analyses; Supplemental Tables 5–7 |  |  |
| **Discussion** | 18 | Summarize key results with reference to study objectives. | Discussion, opening paragraph |  |  |
| Discussion | 19 | Discuss study limitations, considering direction and magnitude of potential bias or imprecision. | Discussion—Limitations | RECORD 19.1: Discuss implications of using data not created for the specific research question, including misclassification, unmeasured confounding, missing data, and changing eligibility over time. | Discussion—Limitations: residual and unmeasured confounding, unavailable diabetes duration and care variables, code-based outcome misclassification, temporal confounding, short follow-up, and observational design. |
| Discussion | 20 | Give a cautious overall interpretation considering objectives, limitations, multiplicity, related studies, and other relevant evidence. | Discussion; Conclusion |  |  |
| Discussion | 21 | Discuss the generalisability (external validity) of the study results. | Discussion—Strengths and Limitations |  |  |
| **Other information** | 22 | Give the source of funding and role of funders. | Declaration—Funding: None | RECORD 22.1: Provide information on how to access supplemental information such as the protocol, raw data, or programming code. | Supplemental Tables 1–7 provide coding algorithms and additional analyses. Patient-level TriNetX data are not publicly shareable because of data-use and privacy restrictions; analyses were conducted within the TriNetX platform. |
